# The YoungFitT project: Study protocol for a randomized mixed-methods trial of physical exercise and mind-body interventions, with or without virtual reality, in university students

**DOI:** 10.1371/journal.pone.0328538

**Published:** 2025-08-01

**Authors:** Samira Rostami, Adrià Bermudo-Gallaguet, Neus Camins-Vila, Blai Ferrer-Uris, Albert Busquets, Mireia Ribera, Laura Coll, Jaime Gallego Vila, Ramon Oliva Martínez, Mel Slater, Gustavo Garcia Diez, Nazareth Perales Castellanos, Mar Larrosa, Jofre Bielsa-Pascual, Pere Torán-Montserrat, Olga Bruna Rabassa, Myriam Guerra-Balic, Kirk I. Erickson, Belinda Brown, Maria Mataró Serrat

**Affiliations:** 1 Department of Clinical Psychology and Psychobiology, University of Barcelona, Barcelona, Spain; 2 Institute of Neuroscience, University of Barcelona, Barcelona, Spain; 3 Sant Joan de Déu Research Institute, Esplugues de Llobregat, Spain; 4 National Institute of Physical Education of Catalonia, University of Barcelona, Barcelona, Spain; 5 Department of Mathematics and Computer Science, University of Barcelona, Barcelona, Spain; 6 Event Lab, Faculty of Psychology, University of Barcelona, Barcelona, Spain; 7 Nirakara Lab, Extraordinary Chair in Mindfulness and Cognitive Science, Complutense University of Madrid, Madrid, Spain; 8 Department of Nutrition and Food Science, Faculty of Pharmacy, Complutense University of Madrid, Madrid, Spain; 9 Metropolitan North Research Support Unit, Jordi Gol i Gurina University Institute Foundation for Primary Health Care Research, Mataró, Spain; 10 Germans Trias i Pujol Research Institute, Germans Trias i Pujol University Hospital, Badalona, Spain; 11 Faculty of Psychology, Education and Sport Sciences Blanquerna, Ramon Llull University, Barcelona, Spain; 12 Advent Health Research Institute, Neuroscience, Orlando, Florida, United States of America; 13 Department of Psychology, University of Pittsburgh, Pittsburgh, Pennsylvania, United States of America; 14 Centre for Healthy Ageing, Health Futures Institute, Murdoch University, Perth, Western Australia, Australia; PLOS: Public Library of Science, UNITED KINGDOM OF GREAT BRITAIN AND NORTHERN IRELAND

## Abstract

**Background:**

Mental health issues among young university students have increased in recent years, driven by academic stress and sedentary lifestyles. The YoungFitT Project aims to explore well-being strategies and the psychobiological mechanisms behind their effects on university students. The project includes two studies: the first evaluates the effectiveness of High-Intensity Functional Training (HIFT), Mindfulness-Based Stress Reduction (MBSR), and Qigong (QG) on psychological well-being and cognitive functions, and also explores whether socio-demographic, mental (mindful thinking, sleep quality), physical (physical fitness, physical activity), physiological (heart rate variability), and biological (microbiota) factors mediate or moderate intervention effects on university students. Given that immersive virtual reality (VR) can enhance adherence and provide additional benefits, the second study will explore the feasibility and efficacy of HIFT-VR, MBSR-VR, and QG-VR on university students’ psychological well-being and cognitive functions.

**Methods:**

Two mixed-methods randomized controlled trials will be conducted. In Study 1, participants will be randomly assigned to one of three groups (HIFT, MBSR, QG) using a 1:1:1 ratio. Psychological, cognitive, physical, physiological, and biological measures will be evaluated two weeks before and after the interventions. The interventions include three weekly sessions for 12 weeks. Subsequently, a follow-up will be conducted 12 weeks after the intervention to assess psychological well-being. Study 2 is a proof-of-concept study in which VR interventions will be co-designed with input from university students and professionals. Twelve participants from each study will also complete semi-structured interviews to explore their experiences and perceived impact.

**Discussion:**

The proposed interventions are expected to produce differential effects on psychological well-being and cognitive function. VR environments may enhance adherence and offer added benefits over conventional training. Findings will inform effective, personalized strategies for the mental and physical health of university youth.

**Trial registration:**

www.ClinicalTrials.gov, identifier NCT06406283; Registration date: 2024/05/06.

## Introduction

Mental health difficulties among youth represent a significant public health concern [1]. A particular focus is on emerging adults aged 18–25, a period that aligns with the onset of many mental disorders; indeed, 75% of such disorders begin before the age of 24 [2]. Notably, approximately 60% of young individuals within this age group are reported to be transitioning into university life, where they face numerous challenges, including academic pressures [3]. During this socially complex and stressful period, university students often engage in unhealthy behaviors, such as insufficient physical activity, potentially leading to a sedentary lifestyle that poses risks to both their mental and physical health [4]. Around one-third of the surveyed first-year university students were identified as likely experiencing at least one mental health disorder, such as anxiety or depression [5]. These mental health challenges can negatively affect various aspects of students’ lives, including emotional well-being, cognitive function, and academic performance. Enhancing cognitive abilities, such as memory and attention, is important not only for learning but also for coping with stress and maintaining psychological balance [6]. Therefore, there is an urgent need to develop and implement mental and physical health-boosting strategies that can be easily incorporated into a student’s daily routine. These strategies could be an effective way for students to manage stress, reduce anxiety, and enhance cognitive awareness, which in turn can lead to better health, productivity, and quality of life [7].

The benefits of physical exercise and mind-body interventions for psychological well-being have been highlighted. Physical exercise has been shown to have positive effects on psychological well-being and cognitive functions, particularly in older adults and children [8,9]. Emerging evidence also suggests similar benefits for young adults and university students [10,11]. As an exercise modality, High-Intensity Functional Training (HIFT) incorporates a mix of intense and varied movements to enhance overall physical condition [12]. HIFT stands out for its intensity, engaging more muscles and requiring greater cardiovascular effort than traditional workouts, while each session takes relatively little time. Its efficient, high-energy demanding format may appeal to busy young adults [12,13]. Growing research findings demonstrate HIFT can improve fitness-related measured in university students. A study comparing CrossFit, a training method similar to HIFT, with traditional weight training in university students found that both improved muscular strength and endurance and helped maintain body composition [14]. Moreover, when comparing High-Intensity Interval Running (HIIT-R) and HIFT in female university students, it was found that HIFT was as effective as HIIT-R in enhancing body composition and aerobic fitness. HIFT led to improved motor performance (sit-ups and standing broad jump), while the HIIT-R protocol showed no improvements [15]. Research on the cognitive and emotional effects of HIFT in university students is still limited. A study on a 10-week online HIFT program during the COVID-19 quarantine found improved reaction times without changes in psychological well-being. Nevertheless, the quarantine adversely affected adherence to the training regimen [16]. To our knowledge, this is the only study that has examined such a program in university students.

Mindfulness-Based Stress Reduction (MBSR) combines various mental and physical practices, such as mindful breathing, body awareness, and yoga, in group settings to promote well-being [17]. MBSR has become a popular tool for addressing the university mental health crisis and is now familiar to many young adults [18]. Substantial evidence supports the effectiveness of mindfulness-based interventions (MBIs) in improving mental health and well-being among university students. Numerous systematic reviews and meta-analyses have demonstrated small to moderate improvements in psychological well-being, particularly in alleviating anxiety, depression, and stress. While these findings are robust, the benefits for other aspects, such as sleep quality, life satisfaction, resilience, mindfulness, subjective well-being, and physical health, are more varied [19–26]. These inconsistencies might stem from differences in study designs, populations, or control conditions. In addition, evidence for cognitive improvements from MBIs in university students is limited, primarily due to the scarcity of studies within this population. A cross-sectional online study suggested that higher levels of mindfulness are associated with better cognitive functions but not with academic achievement [27]. In the context of mindfulness programs, one study reported increased speed in a cognitive flexibility task but no benefits in attention regulation [26]. Another study found no improvement in critical thinking performance following an online mindfulness intervention [28].

In recent years, Traditional Chinese Medicine has gained popularity across Western countries [29]. QG, one of its main branches, utilizes moving meditation practices that emphasize breathing to enhance mental and physical health [30]. QG emphasizes “Qi” (vital energy) and form to harmonize mind and body, promoting physical vitality and mental tranquility [31]. A meta-analysis focusing specifically on university students suggested that QG has the potential to enhance their physical (cardiorespiratory endurance and flexibility) and mental well-being (depression and anxiety). However, no statistically significant effects were observed for muscle strength, vital capacity, heart rate, or mood [32]. Moreover, another meta-analysis on university students with mild to moderate psychological symptoms showed that Traditional Chinese fitness exercises effectively improved depression, anxiety, and sleep disorders [33]. Regarding cognition, although Baduanjin form, one of the most widely practiced types of Health QG, has shown positive effects on executive functions in various populations, with potential benefits for memory, attention, and mental flexibility, there is limited research specifically focusing on university students [34].

Recent meta-analyses comparing different physical exercise and mind-body approaches have reported their specific effectiveness in improving mental health outcomes among young university populations. Subgroup analyses within these reviews reveal mixed results depending on the type of intervention and the specific mental health issue addressed [10,35–39]. For instance, some studies have reported that exercise-based interventions produce the largest effect sizes in improving depression and anxiety among university students [10,36,39], with one indicating that HIIT outperforms other strategies, including QG and Tai Chi [39]. Another study suggests that aerobic exercise may be more effective in reducing anxiety and stress, while traditional Chinese exercises might be particularly beneficial for managing stress [37]. These reviews highlight the need for direct comparisons between different physical exercise and mind-body interventions to better understand their relative effectiveness in addressing mental health outcomes. Notably, no study has directly compared the effectiveness of physical exercise, traditional Chinese practices, and mindfulness-based strategies. Furthermore, a significant gap exists in research comparing the effects of these interventions on cognitive functions in university students.

To better understand the effectiveness of physical exercise and mind-body interventions, exploring their psychobiological mechanisms is crucial. A recent systematic review identified twelve key mediators, including self-efficacy, self-esteem, resilience, and body image satisfaction, which strongly contribute to the positive effects of physical activity on mental health [40]. Among studies focused on university students, one demonstrated that the enduring effects of MBIs on stress are mediated by mindfulness and self-compassion [41]. In addition, another study highlighted the mediating role of self-regulation mechanisms such as attentional control, interoceptive awareness, and emotion regulation in improving young adults’ mental and physical health [42]. Furthermore, a pilot study found that QG’s effects on emotional regulation helped reduce symptoms of depression and anxiety in university students [43]. On the other hand, emerging research has also explored microbiota mechanisms underlying the effectiveness of physical exercise and mind-body interventions in healthy adults, with evidence suggesting a positive relationship between physical activity, cardiorespiratory fitness, and fecal short-chain fatty acid levels [44]. Mindfulness-based cognitive therapy has been shown to enhance bacterial diversity in individuals with high-trait anxiety [45], while practices like Tai Chi and Fitness QG have also been associated with improved intestinal flora health [46]. Despite these findings, the psychobiological mechanisms that explain the emotional and cognitive benefits of physical exercise and mind-body interventions, particularly the relationship between psychological and biological mediators, are poorly understood in young adults.

Translating benefits observed from lifestyle interventions into everyday practices can be challenging [47]. This is particularly evident among university students, who often struggle with maintaining consistent participation and adherence to well-being interventions [48,49]. In this context, technology-based interventions, such as virtual reality (VR) training, may offer an innovative strategy to boost adherence. Immersive VR places users in controlled, artificial environments where key features such as immersion, interaction, and presence can enhance enjoyment, motivation, and engagement, potentially improving treatment adherence [50]. Specifically, improved treatment adherence with MBI-VR has been observed in university students [41]. It has been suggested that VR can also improve long-term exercise adherence [51]. Regarding its effectiveness, VR-based exercise, focusing on traditional aerobic protocols, has shown positive outcomes in improving overall mood states [52] and enhancing working memory function [53] in university students. A systematic review examining VR-based well-being interventions (e.g., MBIs, relaxation, and meditation) in young adults indicated that VR could be an effective method for reducing stress and improving well-being in this population [54]. When compared to conventional mindfulness techniques, VR-based mindfulness training has been found to be more effective in adults [55]. Current research on the effects of VR with different physical exercise and mind-body interventions on psychological well-being and cognition in university students is still limited. Few studies have explored adherence, and none have directly compared their effectiveness. Challenges in this area include the poor use of randomized controlled trial (RCT) designs, variability in VR procedures, and the lack of comprehensive physiological measurements.

In addition to quantitative studies, qualitative research provides deep insights into personal experiences, perceptions, and behaviors. Findings from qualitative and mixed-methods studies have shown that mindfulness interventions improve mental health by reducing anxiety and stress, and by enhancing interpersonal relationships among university students. However, maintaining engagement and long-term practice remains challenging, especially in technology-based interventions [56–59]. Furthermore, QG has shown immediate and long-term benefits, including improved self-awareness, emotional resilience, and a stronger sense of community among students [60]. Several other qualitative studies have also examined mindfulness programs for healthcare students, demonstrating their effectiveness in reducing stress, improving emotional regulation, and enhancing focus. Despite time constraints, many students continued practicing mindfulness informally in their daily lives [61–63]. Qualitative VR studies have also explored mind-body interventions. In a multi-methods design, VR-based meditation enhanced engagement, mindfulness, and focus, with students reporting reduced stress. However, differences in anxiety and depression scores were not statistically significant, likely due to the small sample size [64]. Another study found interactive VR-based mindfulness training effective in improving mindfulness, engagement, and adherence, with the immersive experience boosting motivation and focus. This makes VR a valuable supplement to traditional mindfulness training in high-stress settings [65].

In conclusion, HIFT, MBSR, and QG represent promising well-being strategies. HIFT is an efficient, whole-body physical conditioning modality; MBSR offers a structured and widely accepted method for stress reduction; and QG offers an integrative practice that enhances mind-body harmony. These interventions have demonstrated benefits for psychological well-being and cognitive function in university students. However, to maximize their effectiveness and utilization, it is crucial to investigate the differences and overlap in their effects on various mental health outcomes, as well as to understand their mechanisms of action and the role of individual-level moderators. This can help identify which modality or combination of modalities is most effective in promoting mental well-being during emerging adulthood and may also reveal unique advantages specific to each approach. Furthermore, given their accessibility, cost-effectiveness, and suitability for busy students, these interventions show strong potential to enhance university student well-being, further justifying their study in this context. On the other hand, VR could effectively address issues of low engagement and persistence in training. Given university students’ familiarity with blended learning, they are well-suited for digital mental health support models.

### Objectives and hypotheses

**Fig 1:**
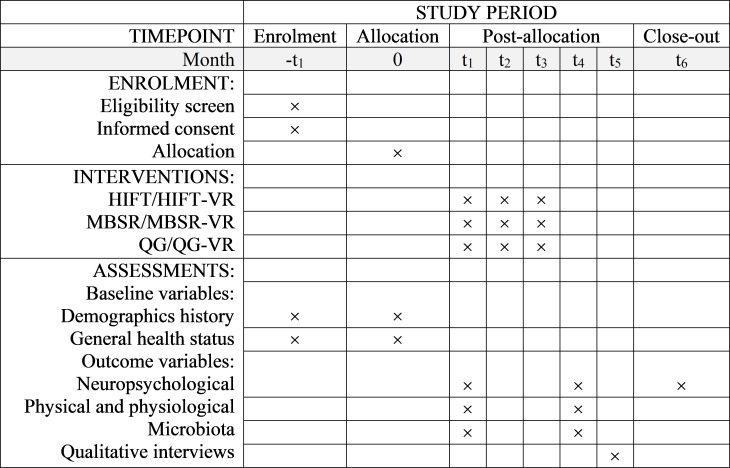
Schedule of enrolment, interventions, and assessments. HIFT: High-Intensity Functional Training, MBSR: Mindfulness-Based Stress Reduction, QG: Qigong, VR: Virtual Reality.

The YoungFitT project, a mixed-methods RCT study, aims to investigate the effects and psychobiological mechanisms of online instructor-led physical exercise and mind-body interventions, as well as their VR-based counterparts, on psychological well-being and cognitive functions. The SPIRIT schedule of enrolment, interventions, and assessments is presented in Fig 1.

**Fig 2:**
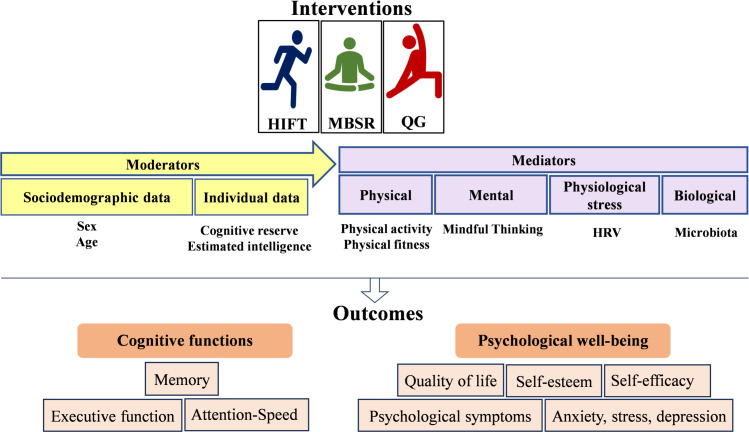
Key variables of interest in the YoungFitT study. HIFT: High-Intensity Functional Training, MBSR: Mindfulness-Based Stress Reduction Therapy, QG: Qigong, VR: Virtual Reality, HRV: Heart Rate Variability.

The project includes two distinct studies:

**Study 1 delves into online physical exercise and mind-body interventions.** Its primary objective is to evaluate the effectiveness of HIFT, MBSR, and QG on the psychological well-being and cognitive functions of university students and to uncover potential mediators and moderators. Specifically, Study 1 aims to explore: (1) the effectiveness of HIFT, MBSR, and QG interventions on the psychological well-being and cognitive functions of university students; (2) the intervention-induced changes in physical aspects (physical activity and fitness status), mental aspects (mindful thinking and sleep quality), and physiological stress markers (heart rate variability [HRV]), and their potential mediating effects on psychological well-being and cognitive outcomes; (3) the intervention-induced changes in the microbiota and its potential mediating effect on psychological well-being and cognitive outcomes; and (4) the potential moderating effects of demographic factors (sex and age) and individual factors (cognitive reserve and general intelligence) on intervention-induced changes in psychological well-being and cognitive outcomes (see Fig 2).

The key hypotheses for this objective are: (1) All three interventions will positively affect psychological well-being and cognitive functions at 12 weeks compared to baseline, with each intervention showing a specific profile of outcomes (HIFT: more cognitive gains; MBSR: more psychological gains; QG: moderate benefits in both areas). These positive effects are expected to persist at the 12-week follow-up; (2) All interventions are expected to induce changes in physical components (increased physical activity and fitness status), mental components (enhanced mindful thinking and sleep quality), physiological stress markers (increased HRV), and microbial composition (increased diversity), which will mediate improvements in psychological well-being and cognitive outcomes; (3) Sociodemographic factors (sex and age) and individual factors (cognitive reserve and general intelligence) will moderate changes in outcomes from pre- to post-intervention.

**Study 2 focuses on VR-based physical exercise and mind-body interventions.** The primary objective of this proof-of-concept study is to test the feasibility and preliminary effectiveness of VR-based HIFT, MBSR, and QG interventions on the psychological well-being and cognitive functions of university students. The specific objectives of this study include: (1) Generating virtual environments for HIFT, MBSR, and QG interventions; (2) Evaluating the usability, acceptability, and feasibility of these VR interventions; (3) Studying the effectiveness of VR interventions as depicted in Fig 2; and (4) Exploring the effects and adherence of these VR-based physical exercise and mind-body interventions compared to non-VR interventions.

It is hypothesized that: (1) All VR interventions will positively affect psychological well-being and cognitive functions after 12 weeks, with each intervention displaying distinct outcomes (HIFT: cognitive gains; MBSR: psychological gains; QG: moderate benefits); and (2) VR-based interventions will yield better adherence and greater benefits compared to non-VR interventions.

### Qualitative objectives and research questions

The qualitative component of the YoungFitT project aims to complement the quantitative findings by exploring participants’ perspectives and insights regarding their experiences with the interventions. The specific qualitative objectives are: (1) to explore participants’ experiences and perceived effects of the HIFT, MBSR, and QG interventions; and (2) to identify barriers and contextual factors influencing participant engagement and adherence to the interventions (e.g., motivational challenges, instructor support, and environmental influences).

The corresponding research questions are: (1) How do participants describe their experiences and perceived benefits from participating in the interventions? and (2) What challenges and contextual factors affect participants’ ability to engage with and adhere to the program?

## Materials and methods

### Study design

**Fig 3:**
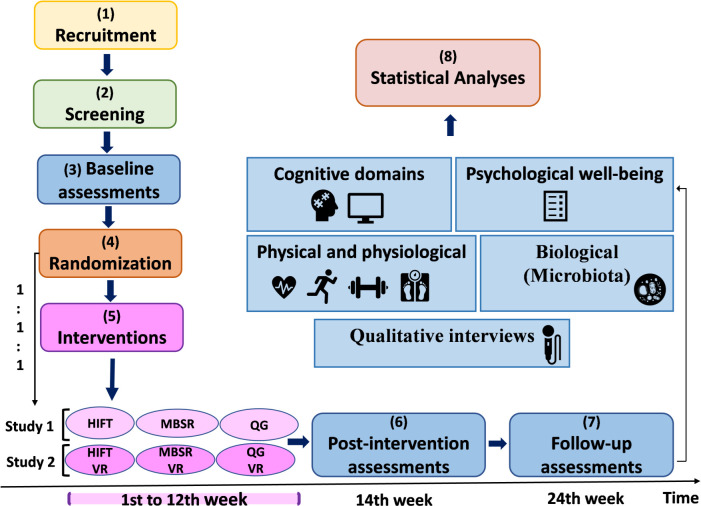
Study design of the YoungFitT project. HIFT: High-Intensity Functional Training, MBSR: Mindfulness-Based Stress Reduction, QG: Qigong, VR: Virtual Reality.

The YoungFitT project is a prospective, three-arm, parallel, single-blinded RCT mixed-methods study planned to be conducted in two phases, as illustrated in Fig 3. Study 1 will begin first, followed shortly by Study 2. Both studies adhere to the SPIRIT guidelines [66] (See S1 File). The Faculty of Psychology at the University of Barcelona will lead this interdisciplinary project, integrating expertise in neuropsychology, sports science, and computer science. The study protocol has been registered at ClinicalTrials.gov (identifier: NCT06406283) and approved by the institution’s Bioethics Committee (IRB number: IRB00003099; protocol code: CER112327) on October 18, 2023 (See S2 and S3 Files). It also complies with the ethical standards outlined in the Declaration of Helsinki. Eligible participants who agree to take part in the study will provide written informed consent. A member of the research team will obtain the signed consent form prior to conducting any baseline assessments. Protocol amendments will be updated on ClinicalTrials.gov and shared with regulatory bodies if necessary.

#### Study status and timeline.

Participant recruitment is anticipated to occur in three waves for Study 1 (February 2024, February 2025, and September 2025) and three waves for Study 2 (September 2025, December 2025, and February 2026), although this schedule may vary depending on study needs. Each recruitment wave will last approximately four months. Recruitment began on 5 February 2024 and is expected to be completed by February 2026. Data collection is actively being conducted and is anticipated to conclude by June 2026. We estimate that results will be analyzed and reported by August 2026.

### Eligibility criteria

Participants will be included in the YoungFitT study if they meet the following inclusion criteria: (1) are university bachelor’s or master’s students, (2) are aged between 18 and 25 years, and (3) have proficiency in either Catalan or Spanish. Participants will be excluded from the study based on any of the following criteria: (1) having injuries that prevent exercise, (2) a history of severe neurological or psychiatric diseases (e.g., schizophrenia or bipolar disorder), (3) a history of alcohol or drug abuse, or (4) conditions causing severe dizziness or related issues affecting VR use (Study 2).

### Recruitment

Participants will be recruited through various strategies, including promotional flyers placed in faculty areas, in-class announcements, email outreach, social media and messaging platforms (e.g., X, Bluesky, Instagram, LinkedIn, and WhatsApp), as well as snowball sampling. To minimize the influence of seasonal or academic stressors (such as exams or holidays), recruitment periods will be strategically scheduled to avoid peak academic times. Interested university students will receive phone verbal explanations regarding the study. Participants will be informed about the study objectives, inclusion and exclusion criteria, the details of study interventions, and their probability of being allocated to each intervention group. Participants will also be informed about the voluntary nature of their involvement and their right to withdraw from the project at any point. Participants who complete the follow-up assessment will receive compensation of 30 euros.

### Randomization

In Study 1, an independent researcher will use a software-generated random sequence to allocate participants into three groups at a 1:1:1 ratio for each wave (Fig 3). The randomization list will be securely generated and stored on a university server. While the research team will be aware of the allocation sequence, participants will remain unaware of their group assignment until the intervention. In Study 2, participants will be assigned to one of three intervention groups through sequential cohort assignment based on recruitment order. Potential biases in group composition will be mitigated by monitoring baseline characteristics across groups and adjusting for these in subsequent analyses. Outcome assessors will be blinded to group allocation through the use of coded identifiers to ensure unbiased assessments. To prevent unintentional disclosure, participants will be reminded not to discuss their group allocation during post-intervention assessments. To assess blinding success, outcome assessors will guess participants’ group assignments after the final evaluation. The proportion of correct guesses will be compared to the 33% chance level using a chi-square goodness-of-fit test with one degree of freedom. Data analysts will conduct analyses without access to group allocation information, thereby ensuring the integrity of the results.

### Sample size

A power analysis was conducted using G*Power software (version 3.1.9.4) for a one-way ANOVA (fixed effects, omnibus) with three groups. Assuming a medium effect size (f = 0.25), α = 0.05, and a desired power of 0.80, the analysis yielded a required sample size of 159 participants, with a critical F-value of 3.054. To accommodate an expected dropout rate of 10%, the sample size was adjusted using the proportional method, resulting in a final target of 177 participants (59 per group). This adjustment ensures that statistical power remains adequate even if participant attrition occurs. Study 2 will include 45 participants (n = 15 per group) as part of a proof-of-concept trial intended for preliminary exploration. For Study 1, biological samples (fecal) will be collected from a randomly selected subgroup of 90 participants (30 per group) before and after the interventions. Furthermore, a subset of 24 participants (12 from each study) will be selected for the qualitative study using purposive sampling to capture diverse perspectives [67]. The sample size is based on previous studies [56,61,68] and evidence suggesting that qualitative inquiry often reaches data saturation with approximately 12 participants [69].

### Interventions

#### Online physical exercise and mind-body interventions.

The training program will run for 12 weeks, consisting of three sessions per week, all conducted remotely. Participants will take part in weekly synchronous sessions in groups of 15 and will also complete autonomous sessions on days of their choice. Sessions will be conducted using a secure version of Zoom with end-to-end encryption and privacy settings, following recommended standards for technology-based health interventions [70]. An orientation session will be held at the beginning to introduce the program, weekly schedules, required materials, safety guidelines, proper exercise techniques, training on the Zoom platform, and a question-and-answer segment. To participate in the training sessions, participants will need the following materials: (1) a digital device (computer, tablet, or mobile phone) with an internet connection, camera, and microphone; (2) basic training items, including an exercise mat, armchair or sofa, towel, full water bottle, and comfortable workout attire, depending on the specific interventions; and (3) additional materials, which will be outlined in the following sections. With participants’ prior consent, Zoom sessions led by instructors will be recorded to facilitate participant review and repetition, and also to support training quality assurance and research purposes. These recordings will be securely stored on university-hosted cloud storage. Each group will be encouraged to use a dedicated WhatsApp group to connect with one another and the research team, fostering an environment conducive to sharing experiences.

#### High-intensity functional training intervention.

HIFT will consist of two 60-minute online group synchronous sessions, which will be recorded, and one individual autonomous session each week, during which participants can repeat a session of their choice from that week. Participants will need dumbbells or alternative items (e.g., water bottles, soda cans, or milk cartons) as weights for certain strength exercises, as well as a chair for support during specific movements. They will also receive a document explaining the Borg Scale (range: 6–20), with detailed descriptions of each score to assist them in monitoring effort during training [71]. The sessions will be organized as follows:

(1) Introduction (5 minutes): Welcome to the session and an overview of the training schedule, (2) Warm-up (10 minutes): Preparation for the exercise session through joint mobility exercises, dynamic stretching, and whole-body activities to elevate the heart rate, (3) Technique (10–20 minutes): Explanation and practice of proper exercise form for the movements included in the main session, with special attention to new exercises. This will be followed by an explanation of the specific type of HIFT to be performed that day, (4) Workout (15–25 minutes): The main portion of the session, consisting of HIFT drills, and (5) Cool-down (10 minutes): A gradual return to normal physiological levels through stretching exercises [12]. For the HIFT block, three distinct workout models commonly used in CrossFit training will be utilized and combined throughout the weeks: (1) Every Minute on the Minute (EMOM): Participants complete the prescribed number of repetitions for each exercise within one minute and use the remaining time for recovery, (2) As Many Rounds as Possible (AMRAP): Participants perform as many rounds as possible of the prescribed exercises and repetitions within a set time limit, and (3) For Time (FT): Participants complete the prescribed exercises and repetitions in the shortest time possible, within a predetermined time cap [72]. Table 1 presents the HIFT protocol. Intensity can be scaled by adjusting the number of repetitions and load, modifying the range of motion, or extending rest periods as needed. As participants adapt to the training, progression will be achieved by gradually increasing the complexity, intensity, or volume of the workouts [73]. The program will be led by an instructor with a degree in physical activity and sport sciences, who has experience in implementing physical exercise programs for young adults.

**Table 1:** High-intensity functional training (HIFT) protocol.

Week	Session	Type of session	Duration (min)	Sets	Exercises × Reps/ Time
**1**	1	EMOM	15	5	Push-ups × 15, Air squats × 20, Plank × 30 Seconds
	2	FT	15	4	Fons triceps × 20, Lunge × 40, Burpees × 20, Mountain climbers × 40
**2**	1	EMOM	15	5	Fons triceps × 15, Lunge × 24, Jumping jacks × 35
	2	AMRAP	15	∞	Thrusters × 20, Burpees × 20, Push-ups × 20
**3**	1	EMOM	20	5	Push-ups × 15, Air squats × 20, Burpees × 20, Sit-ups × 15
	2	FT	20	4	Fons triceps × 20, Lunge × 40, Burpees × 20, Mountain climbers × 40, Thrusters × 30
**4**	1	EMOM	20	5	Push-ups × 15, Hip thrust × 30, Jumping jacks × 35, Plank × 30 seconds
	2	AMRAP	20	∞	Shoulder press × 20, Burpees × 20, Push-ups × 20, Air squats × 40
**5**	1	EMOM	25	5	Fons triceps × 15, Hip thrust × 30, Burpees × 15, Plank × 40 seconds, Jumping jacks × 40
	2	AMRAP	25	∞	Shoulder press × 20, Burpees × 20, Sit-ups × 20, Air squats × 20
**6**	1	EMOM	25	5	Push-ups × 20, Lunge × 30, Skipping × 40, Hip thrust × 20, Mountain climbers × 30
	2	FT	25	4	Thrusters × 40, Push-ups × 40, Air squats × 40, Sit-ups × 40
**7**	1	EMOM	15	5	Pike push-ups × 15, Squat leg rises × 20, Spider plank × 40
	2	FT	15	4	Bent-over row × 20, Lateral lunge × 40, Skipping × 40, V-ups × 20
**8**	1	EMOM	15	5	Fons triceps × 15, Lateral lunge × 24, V-ups × 25
	2	AMRAP	15	∞	Bent-over row × 20, Sumo squat × 20, Pike push-ups × 20
**9**	1	EMOM	20	5	Pike push-ups × 15, Lateral lunge × 30, Spider plank × 40, Burpees × 10
	2	FT	20	4	Bent-over row × 20, Squat leg raises × 40, Jumping jacks × 35, V-ups × 20, Thrusters × 30
**10**	1	EMOM	20	5	Hand release push-ups × 15, Hip thrust × 30, Skipping × 40, Lateral lunge × 30
	2	AMRAP	20	∞	Alternating hand plank × 15, Sumo squat × 40, Pike push-ups × 20, V-ups × 30
**11**	1	EMOM	25	5	Pike push-ups × 20, Sumo squat × 20, Plank × 40 seconds, Burpees × 20, Skipping × 40
	2	AMRAP	25	∞	Bent-over row × 20, Squat leg raises × 20, Hand release push-ups × 20, Sit-ups × 20
**12**	1	EMOM	25	5	Pike push-ups × 20, Lateral lunge × 30, Alternating hand plank × 15, Jumping jacks × 40, Spider plank × 40
	2	FT	25	4	Hand release push-ups × 20, Sumo squat × 40, V-ups × 40, Thrusters × 40

EMOM, Every minute on the minute; FT, For time, AMRAP, As many rounds as possible;

∞, Infinite.

#### Mindfulness-based stress reduction intervention.

The program will comprise a 120-minute online synchronous session and two individual practice sessions per week, each lasting 20–40 minutes. Participants will receive a coursebook aligned with each class session, featuring: (1) a session introduction, (2) theoretical concepts, (3) key practical themes, (4) a weekly practice guide, (5) additional reading materials, and (6) visual resources. Audio recordings will also be provided in MP3 format. The applied method will follow the official MBSR program guidelines developed by Jon Kabat-Zinn [74], with some adaptations based on our previous work [75]. The program will be extended to 12 weeks to align with the HIFT and QG programs. Key changes include: (1) Session 0: A 2-hour introductory session featuring an overview, distribution of materials, and the basics of mindfulness practice, (2) An all-day practice session of approximately 6 hours, scheduled between Sessions 6 and 7, (3) Two unscheduled weeks (between Sessions 3 and 4, and between the practice day and Session 7), during which participants will be encouraged to maintain autonomous practice using the provided materials and recordings. The program will be led by a certified mindfulness instructor affiliated with the MBSR Professional Association of Mindfulness Instructors.

#### Qigong intervention.

Like HIFT, the QG intervention will consist of two 60-minute weekly online synchronous group sessions and one individual autonomous session, giving participants the opportunity to revisit any session of their choice from that week. In addition to Zoom, Open Broadcaster Software (OBS) will combine music, PowerPoint slides, and live broadcasts. Participants will be asked to find a peaceful space with fresh air, ideally measuring 3m x 2m and free of objects. The sessions will consist of three parts: (1) Warm-Up: The “Three Gates” exercise and the “Five Harmonies” of Liu Ya Fei, focusing on spine and joint strengthening; (2) Main Part: The 8 Qigong exercises from the Standardized Baduanjin Qigong for Health, as instructed by the Chinese Health Qigong Association [76]. This includes reviewing previous exercises, joint practice, and supervised practice; (3) Self-Massage: A selection of self-applied Tui-na massages. The training program will be led by two qualified and certified instructors from the Qigong Institute of Barcelona. One will primarily demonstrate the exercises, while the other provides explanations and monitors participants’ performance. The dual-instructor format is considered beneficial for ensuring safety and clarity in instruction, which emphasizes precise postural alignment and breathing. Efforts will ensure consistency in instructional content and participant interaction across all interventions.

#### Virtual reality-based physical exercise and mind-body interventions.

The immersive VR system will be co-designed through a structured focus group involving six participants, consisting of university students and professionals. The focus group aims to explore the integration of well-being interventions into VR environments. One session will be conducted online via Zoom and will last approximately 60 minutes. A moderator will guide the discussions using a structured interview script (S4 File). The discussions will revolve around key questions concerning the feasibility, advantages, and challenges of implementing these practices in a VR environment. The sessions will be recorded using Zoom’s built-in recording feature, with participants’ prior consent. The recordings will then be transcribed and analyzed thematically. All responses will remain anonymous, and data will be securely stored on the university’s server. Session characteristics (e.g., frequency, duration) will be informed initially by existing literature on VR-based well-being interventions and further adapted throughout the co-design process to determine the optimal configuration for delivering VR-based interventions. Expert evaluations and usability assessments will ensure that the intervention meets user needs and well-being objectives. A computer science technician will develop the VR environments using Unity, a widely used real-time 3D development engine [77]. These environments will feature realistic avatars representing the participants. The proficient instructors who conducted the training in Study 1 will also be involved in developing the VR programs for Study 2. Participants will be provided with Meta Quest 3S headsets (Meta) to train remotely, fully utilizing the potential of VR through embodiment, body tracking, music, and immersive environments such as natural settings.

### Intervention adherence

In Study 1, attendance for online classes will be recorded and tracked by instructors using Excel spreadsheets. Participants will self-report their independent practice sessions using a questionnaire administered via Microsoft Forms. This will capture the frequency and duration of autonomous practice. Furthermore, participants will have the option to provide comments on their experiences. A researcher will review these records weekly, verify the accuracy of the data, and update instructors on participants’ training status. Adherence will be assessed based on session attendance and the total time spent on both supervised and independent practice. Participants in Study 2 will also record their attendance in a Microsoft Forms questionnaire, which will subsequently be reviewed by a researcher. Adherence will be based on session attendance and VR training duration.

### Safety measures

At the start of the intervention, participants will receive instructions on how to prevent potential injuries and manage discomfort. For Study 2, safety measures will also include considerations for risks associated with VR use, such as motion sickness and user fatigue. Participants will be encouraged to report any adverse events at any point during the study and to document any difficulties or discomfort experienced during or after the sessions in a questionnaire. These questionnaires will be reviewed weekly by the instructors. Any serious incident (e.g., significant injury or medical event) will be reported immediately to the Principal Investigator and, if necessary, to the institutional ethics committee. The incident will be reviewed to determine necessary protective actions. Participants may discontinue or modify interventions upon request or due to adverse events.

### Confidentiality and data access

Each participant will be assigned a unique alphanumeric code based on their order of inclusion to ensure confidentiality and privacy throughout the study. The Principal Investigator and Project Manager will maintain a confidential record linking each participant to their assigned code. All trial data will be anonymized and stored in a secure university database dedicated to the project, accessible only to authorized members of the research team. Once the database is complete, it will undergo a quality control process to verify its accuracy and integrity.

### Assessments

Quantitative data will be collected first to assess the effectiveness of the interventions and to explore potential mechanisms of change. Assessments will include psychological, cognitive, physical, and physiological measures, conducted within two weeks before the start of the interventions and within two weeks after their completion. A 12-week follow-up will be conducted through online questionnaires to assess long-term psychological well-being. As part of Study 1, biological data will be collected through fecal samples obtained before and after the interventions.

### Baseline measures

After signing the informed consent form, eligible participants will complete baseline questionnaires. These questionnaires will gather demographic information, including age, relationship status, field of study, university, academic program, academic performance, employment status, socioeconomic status, and health history, as part of the demographics and health history questionnaire. Additional questionnaires will assess cognitive reserve [78], personality traits [79], and participants’ expectations regarding treatment outcomes. To estimate general intelligence, the Vocabulary and Matrix Reasoning subtests from the Wechsler Adult Intelligence Scale–III (Wechsler, 2001) will be included in the baseline neuropsychological evaluation [80].

### Outcome measures

#### Psychological well-being.

Depression, anxiety, and stress will be assessed using the Depression Anxiety Stress Scale (Table 2), with changes in scores serving as the primary outcome measures [81]. Additional aspects of psychological well-being will be evaluated through multiple questionnaires assessing psychological symptoms [82], self-esteem [83], mindfulness [83], self-efficacy [84], and quality of life [85] (Table 2).

**Table 2:** The proposed assessments of YoungFitT.

Outcome measures	Measure method	Description
**Psychological**		
Psychological Symptoms	90 Symptoms Inventory (90-SCL-R)	Direct scores: 0–4; higher scores reflect more symptomatology.
Self-esteem	Rosenberg Self-Esteem Scale (RSE)	Direct scores: 10–40; higher scores indicate higher self-esteem
Depression, Anxiety, and Stress	Depression, Anxiety, and Stress Scale-21 (DASS-21)	Direct score: 0–126; higher scores represent more global symptoms
Mindfulness	Five-Facet Mindfulness Questionnaire (FFMQ)	Direct scores: 39–195; higher scores reflect more mindfulness levels
Self-efficacy	General Self-Efficacy Scale (GSE)	Direct scores: 10–40; higher scores indicate more self-efficacy
**Cognitive**		
Immediate verbal attention	Direct Digit Span (WAIS-III)	Direct score: 0–9; higher scores indicate better performance
Processing speed	Symbol-Digit Coding (WAIS-III)	Direct score: 0–133; higher scores indicate better performance
Visual attention	Trail Making Test Part A	Time (seconds) to complete the numerical sequence. Direct score; higher scores represent poorer performance
Verbal memory	Rey Auditory Verbal Learning Test	Direct score: 0–75; higher scores reflect better performance
Visual memory	Rey-Osterrieth Complex Figure	Memory drawing accuracy at 3–5 minutes. Direct score: 0–36; higher scores indicate better performance
Executive Function – Flexibility	Trail Making Test Part B	Time (seconds) to complete the alphanumeric sequence. Direct score; higher scores reflect worse performance
Executive Function – Inhibition	Stroop Color and Word Test	Interference score calculated as CW − ((W × C/ (W + C)). Higher scores represent better performance. Negative values indicate poorer performance
Executive Function – Verbal Fluency	Phonetic Fluency: Controlled Oral Word Association Test and Semantic Fluency with “Animal” category	Total words evoked in 60 seconds for each letter (P, M, R) and animals. Direct score; higher scores indicate better performance
Verbal Digit Working Memory	Backward Digit Span (WAIS-III)	Span. direct score: 0–8; higher scores reflect better performance
Verbal Comprehension	Vocabulary subtest (WAIS-III)	Direct score: 0–66; higher scores indicate better performance
**Lifestyle**		
Sleep Quality	Pittsburgh Sleep Quality Index (PSQI)	Direct score: 0–21; lower scores reflect better sleep quality
Physical Activity	International Physical Activity Questionnaire (IPAQ)- Short Form	Total physical measured in MET-min/week. Higher scores indicate more physical activity.
Diet	Mediterranean Diet Assessment Tool (PREDIMED)	Direct score: 0–14; higher scores indicate more adherence to a Mediterranean diet
	93-item Food Frequency Questionnaire (FFQ)	Estimate the usual intake of nutrients and food groups over the past 12 months
	Bristol Stool Form Scale	Classifies stool into 7 types based on form and consistency; indicates gut health
**Physical and Physiological**		
Height	Wall-mounted tape measure	Stand upright, feet flat, heels together, head neutral; in meters (m)
Weight	Digital scale	Stand on the scale without shoes; in kilograms (kg)
Body Mass Index (BMI)	Formula: BMI = weight (kg)/ height (m)^2^	Calculate using weight in kg and height in meters squared
Waist and Hip Circumference	Flexible measuring tape	Measure at the natural waistline and at the widest part of the hips in centimeters (cm)
Waist-to-Hip Ratio (WHR)	Formula: WHR = waist circumference (cm)/ hip circumference (cm)	Calculate using waist and hip circumferences in cm
Aerobic Capacity	Submaximal oxygen consumption test	Estimated VO_2_ based on submaximal step test (ml/kg/min)
Lower Body Muscle Power	Countermovement jump (CMJ)	Velocity and power measured in meters per second (m/s)
Strength of the Hand Flexor Muscles	Han-grip test	Right and left hand grip strength in kilogram-force
Balance	Y-balance test	Composite score of each leg Y Balance (% of leg length). Formula: ((YBALANCE_ANTERIOR + YBALANCE_POSTEROMEDIAL+ YBALANCE_POSTEROLATERAL/ (3 × LEG_LENGTH)) ×100
Flexibility	Sit-and-reach test	Sit with legs extended, reach forward along the measuring scale; in cm
Heart Rate Variability (HRV)	Root mean square of successive differences (RMSSD) method	Measure variation in time between successive heartbeats
Systolic and Diastolic Blood Pressure	Sphygmomanometer	Measure with cuff and pressure gauge; in millimeters of mercury (mmHg)
**Biological**		
Microbiota composition and functionality	16S *rRNA* gene sequencing	Sequence of the V3-V4 16S *rRNA* gene regions and metabolic inference
**Perceived outcomes and qualitative interviews**		
Expectation questionnaire	Assesses perceived effects of the intervention on cognition, well-being, fitness, and quality of life	
Semi-structured interviews	Twelve participants per group will report perceived impact, benefits, and barriers to engagement	
**Usability Virtual Reality System**		
System Usability Scale (SUS)	Direct scores: 0–100; higher scores indicate better usability	

#### Cognitive functions.

The battery tests were grouped into four cognitive domains (Table 2), based on the theoretical framework proposed by Lezak et al. (2012) and Strauss and Spreen (1998). Composite scores for each domain will be derived by converting raw test scores into Z-scores, standardized using the baseline mean and standard deviation. To ensure consistency across assessments, all Z-scores will be referenced to the baseline distribution. Domain-specific composite scores will be calculated by averaging the Z-scores of individual tests within each domain. A global cognitive function score will then be computed by averaging these domain-level composites, providing an overall measure of cognitive performance. Changes in cognitive domains and global cognition will be considered primary outcome measures [86,87].

#### Lifestyle.

Physical activity [88], sleep quality [84], adherence to the Mediterranean diet [89], dietary intake [90], and gut health [91] will be assessed as lifestyle-related variables. Detailed descriptions of these measures are provided in Table 2.

#### Physical and physiological measures.

Anthropometric characteristics, including height, weight, and waist and hip circumferences, will be assessed following the procedures recommended by the International Society for the Advancement of Kinanthropometry (ISAK). Body Mass Index (BMI) will be calculated from height and weight measurements and Waist-to-Hip Ratio (WHR) will be calculated from waist and hip circumference [92]. The assessment will also include physical fitness components such as aerobic capacity [93], balance [94], flexibility [95], handgrip strength [96], and lower body muscle power [97]. Physiological stress will be examined through HRV, analyzed using the root mean square of successive differences (RMSSD) method [98], along with measurements of systolic and diastolic blood pressure in millimeters of mercury (mmHg) [99]. A detailed overview of all assessments is provided in Table 2.

#### Biological measure.

Stool samples will be collected using the DANASTOOL Sample Collection MICROBIOME Kit (Danagen-Bioted, BCN, Spain). Participants will receive instructions and will be asked to collect stool from 2–3 different spots using the attached spoon, then transfer the sample into the stabilizing liquid in the tube to preserve microbial DNA. After the sample is added, the tube will be shaken to ensure homogenization. Each tube will be labeled with the participant’s code and collection date before being sent to the laboratory at room temperature (15–25 °C) for analysis. Microbial DNA will be extracted from a 0.5–1.0 g stool sample using the DANAGENE MICROBIOME Fecal DNA Kit, following the manufacturer’s protocol [100]. The microbial composition will be assessed through 16S *rRNA* gene-targeted sequencing. The sequencing data will then be processed and analyzed using the ZymoBIOMICS Service: Targeted Metagenomic Sequencing (Zymo Research, Irvine, CA). To complement taxonomic profiling, we will conduct a functional prediction analysis of the gut microbiota using *Phylogenetic Investigation of Communities by Reconstruction of Unobserved States,* version 2 (PICRUSt2) [101]. This tool infers the potential metabolic functions of microbial communities based on 16S *rRNA* gene sequencing data and reference genome databases. After quality control and OTU/ASV assignment, functional profiles will be predicted in terms of gene family abundance (e.g., KEGG Orthologs) and metabolic pathways (e.g., KEGG pathways, MetaCyc).

#### Usability and feasibility of VR interventions.

The quantitative assessment of the VR system’s usability will be conducted using the System Usability Scale (SUS), with scores ranging from 0 to 100, where higher scores indicate greater usability [102]. The feasibility of the VR program will be assessed through the following: (1) Measuring engagement levels, based on adherence rates, including session attendance and time spent in VR; (2) Evaluating program acceptability by collecting participant feedback through structured interviews, guided by the Unified Theory of Acceptance and Use of Technology (UTAUT), to better understand their experience with the VR intervention; (3) Analyzing effectiveness, using outcome measures relevant to the study objectives.

#### Perceived outcomes and qualitative interviews.

Participants will complete an expectation questionnaire to assess their perceived treatment effects on cognition, emotional well-being, physical fitness, and quality of life resulting from the study interventions. A purposive subsample of twelve participants from each study will also be invited to participate in a semi-structured interview to explore their experiences with the intervention and their perceptions of its impact (see S5 File). These interviews will examine perceived benefits, barriers, and contextual factors that influenced participant engagement and adherence, such as motivation and environmental influences. Participants will also be encouraged to reflect on their overall experience and offer recommendations for improving future interventions. All interviews will be audio-recorded and transcribed verbatim, and the transcripts will undergo participant validation to enhance accuracy and completeness. All data will be securely stored on a password-protected institutional server, in accordance with institutional data protection and retention policies. Furthermore, documentation will be maintained throughout the data collection phase to enhance transparency and consistency [103]. The qualitative data will be integrated with the quantitative findings using a complementary mixed-methods approach to provide a more comprehensive understanding of the intervention’s effects [104].

### Statistical analyses

Data will be entered into a version-controlled Excel database. Analyses will be conducted using the latest available versions of IBM SPSS Statistics and R. A two-tailed p-value < 0.05 will denote statistical significance, with adjustments for multiple comparisons applied where appropriate. Descriptive statistics (means, standard deviations, medians, ranges, and frequencies) will summarize baseline characteristics overall and by group. We will assess distributional assumptions, including skewness and the presence of outliers. Missing data will be characterized by reporting the percentage of missing values for each variable and by evaluating the most likely mechanisms, classified as missing completely at random (MCAR), missing at random (MAR), or missing not at random (MNAR).

Primary hypotheses will be tested under an intention-to-treat (ITT) framework, including all randomized participants. Analysis of covariance (ANCOVA) will model change scores (post-intervention – baseline) using group, baseline score, and selected covariates as predictors. Sex and age will be included as covariates in all analyses. Additional covariates will be considered from a pool of theoretically relevant candidates if they show a significant and meaningful association with the outcomes. To minimize multicollinearity, we will examine the interrelationships among selected predictors and retain only one variable when substantial overlap is observed. If the omnibus test is significant, we will perform pairwise comparisons within each outcome variable using appropriate corrections for multiple comparisons (e.g., Bonferroni). Assumptions of homogeneity of regression slopes, normality, and homoscedasticity of residuals will be checked. If violated, alternative models (e.g., mixed-effects models for repeated measures) will be considered. Between-group findings will be supplemented by within-group analyses (paired t-tests or Wilcoxon tests, as appropriate).

Missing outcome data will be handled using multiple imputation by chained equations (MICE), assuming data are MAR. Predictive mean matching will be applied, with the number of imputations and iterations guided by current recommendations [105]. The imputation model will mirror ANCOVA predictors and include auxiliary variables to improve accuracy. We will assess imputation diagnostics (trace plots, distributional comparisons) and pool results using Rubin’s rules. Complete-case analyses and sensitivity tests under MNAR will also be reported. Exploratory analyses will examine adherence as a continuous predictor and test adherence-by-treatment interactions. These models will include the same set of covariates used in the primary ANCOVA analyses to ensure comparability. Mediation and moderation effects will be tested using Hayes’s PROCESS macro with a minimum of 5,000 bootstrap samples. Given the proof-of-concept nature of Study 2, effect sizes (partial eta squared for ANCOVA) will be reported to aid interpretation of the findings.

### Qualitative data analysis

We will conduct thematic analysis to systematically identify, code, and analyze themes and subthemes emerging from the interview data [105]. The analysis will focus on participants’ experiences and perceptions of the interventions. NVivo software will be used for data organization and coding. To enhance analytical rigor, independent coding will be conducted by at least two researchers, with discrepancies resolved through discussion. Representative quotes will be selected to illustrate key themes, ensuring that findings are grounded in participants’ narratives. Data will be interpreted in relation to the broader study objectives, providing insights into participants’ perspectives on the effects of the intervention.

## Discussion

The YoungFitT project is an RCT designed to explore the effects and psychobiological mechanisms of physical exercise and mind-body interventions, including virtual reality (VR) strategies, on the psychological well-being and cognitive functions of university students. To effectively implement these therapies, we address three fundamental questions: (1) How do different levels of mind-body engagement within interventions affect various cognitive and psychological well-being outcomes? (2) What potential mediators and moderators influence the effectiveness of these strategies? (3) What factors motivate young adults to participate in these training programs?

Each intervention is expected to enhance psychological well-being and cognitive functions. We anticipate that the MBSR group will show the most significant psychological improvements, the HIFT group will demonstrate the greatest cognitive benefits, and the QG group will offer balanced mental and physical benefits. Secondly, our focus is on the psychobiological mechanisms underlying physical exercise and mind-body programs. We expect to observe changes in physical and mental measures and the microbiota, which may vary depending on the intervention. In addition, understanding how sociodemographic and individual factors moderate these changes is essential. This knowledge is critical for developing personalized well-being strategies tailored to individual students during their education and early career years. As future leaders and decision-makers, university students’ improved well-being may influence future policies on mental and physical health across diverse settings. In the final stage, we will assess the feasibility and effectiveness of VR-based physical exercise and mind-body interventions, aiming to enhance adherence and improve psychological well-being and cognitive functioning in university students. Integrating VR into interventions may offer additional benefits, promote physical activity, and serve as a preventive health tool. This represents a positive step forward in the development of effective, immersive, and advanced training methods to support mental and physical health in university students and broader populations.

Certain practical and operational issues in executing the YoungFitT project require discussion. First, recruiting and retaining participants, as well as ensuring adherence to intervention protocols, presents significant challenges. Engaging university students, who often have busy schedules and exam-related commitments, requires strategic planning and effective communication. To address recruitment challenges, we will implement alternative strategies, including outreach to additional universities and faculties. Our approach also includes offering flexible scheduling options and collecting detailed information about participants’ experiences and any obstacles they encounter in maintaining engagement. Furthermore, as Study 2 is an exploratory proof-of-concept study, the relatively small sample size may limit the ability to detect statistically significant between-group differences. Therefore, the findings will be considered preliminary and are primarily intended to inform the design and implementation of future, larger-scale investigations. Finally, the implementation of VR-based interventions may present technical difficulties. Technical support from IT personnel and timely equipment replacement will help mitigate these challenges.

In conclusion, our goal is to ensure the smooth execution of the YoungFitT project and to maximize its impact on enhancing the mental and physical health of students through innovative physical exercise and mind-body interventions. This effort aims to provide valuable insights that can guide future approaches to promoting student well-being throughout their academic and professional journeys.

## Dissemination

The results of the project will be disseminated through peer-reviewed journals and international scientific conferences in sport sciences, neuroscience, and VR. They will also be shared with students via university communication channels, health promotion services, and student associations, as well as with the general public through dedicated platforms (e.g., social media).

## Supporting information

S1 File SPIRIT checklist. (PDF)

S2 File Study protocol approved by the Bioethics Commission of the University of Barcelona (Original version). (PDF)

S3 File Study protocol approved by the Bioethics Commission of the University of Barcelona (English version). (PDF)

S4 File Structured focus group interview guide for VR well-being interventions. This focus group guide is used to explore user experience, technical considerations, motivation, and feedback mechanisms related to integrating HIFT, QG, and mindfulness into VR. The guide includes thematic sections and open-ended questions to support in-depth discussion and user-driven design.
(PDF)

S5 File Semi-structured interview guide. This interview guide is designed to explore participants’ experiences, perceptions, and reflections related to the intervention. It includes open-ended questions covering engagement, motivation, perceived effects, barriers, and long-term impact.
(PDF)
