# Supplementary material for: The YoungFitT project: Study protocol for a randomized mixed-methods trial of physical exercise and mind-body interventions, with or without virtual reality, in university students
Source: PLoS One. 2025 Aug 1;20(8):e0328538. doi: 10.1371/journal.pone.0328538 (PMC12316210; doi:10.1371/journal.pone.0328538)
Supplement: S2 File — (PDF) [file pone.0328538.s002.pdf]

UNIVERSITAT DE BARCELONA

COMISSIÓ DE BIOÈTICA

FORMULARI 1

Declaració d'existència d'experimentació amb éssers humans o de la utilització de mostres biològiques d'origen humà

[X] El/La sotasignant, investigador/a principal del Projecte titulat:

Joves actius, ment sana: Estratègies per a la salut cerebral i el benestar psicològic en joves: un estudi aleatoritzat de mètode mixt amb realitat virtual i sense realitat (YoungFitT: Young Fitness Technology).

Declaro que:

[X] Sí implica experimentació amb humans

[X] Sí s'utilitzaran mostres d'origen humà

Nom i cognoms: Maria Mataró Serrat
Departament / Unitat: Departament de Psicologia Clínica i Psicobiologia / Secció Psicobiologia
Adreça postal: Pg Vall d'Hebron, 171

Signatura i data:

20 de setembre de 2023

UNIVERSITAT DE BARCELONA

COMISSIÓ DE BIOÈTICA

Documentació que s'ha d'adjuntar:

En tots els casos: Breu justificació científica i ètica del projecte de recerca que inclogui una descripció de l'impacte que es preveu sobre les persones participants i dels beneficis i riscos que es preveuen com a resultat de la recerca.

A) Si ja es disposa de l'informe **favorable** del Comitè Ètic d'Investigació del centre en el qual es portarà a terme l'experimentació o que subministrarà les mostres, cal adjuntar-ne una còpia escanejada.

B) En altres supòsits, cal adjuntar:

· Informació detallada que rebrà el voluntari sotmès a les proves. En els casos en què es prevegi sol·licitar la participació de menors d'edat legal en projectes de recerca o tesis de doctorat, caldrà que en el full de consentiment, a més de la signatura dels pares o responsables legals dels menors, hi consti de manera explícita l'assentiment dels dits menors i que se'ls hagi informat prèviament, atenent a criteris de gradualitat en la seva capacitat de comprensió, de les característiques, procediments i finalitat del projecte. A més, l'equip investigador vetllarà perquè al llarg de tot el procés d'experimentació es tingui molt en compte el benestar i el comfort del menor, amb atenció especial al possible rebuig o negativa del menor a seguir participant en el projecte.

Sobre protecció de dades personals:

- Atès el que estableixen el Reglament 2016/679 de la Unió Europea, de 27 d'abril, relatiu a la protecció de les persones físiques en relació al tractament de les seves dades personals i la Llei Orgànica 3/2018, de 5 de desembre, de protecció de dades personals i garantia dels drets digitals, en el cas que en la realització del projecte de recerca o la tesi de doctorat s'obtingui dades personals -incloses dades biomètriques, genètiques i quasevol altra de caràcter personal i amb especial cura quan es tracti d'infants-, cal fer constar explícitament qui és el responsable del tractament i custòdia de les dades recollides, així com en quin dispositiu s'emmagatzemaran i/o es procesaran i de quin tipus de dades es tracta: si són codificades, seudonimitzades o anonimitzades.
- A més a més, tant els investigadors sèniors com els estudiants de doctorat hauran d'afegir als fulls d'informació als voluntaris participants i de consentiment els paràgrafs següents:

  a) De conformitat amb l'establert a l'esmentada regulació, la UNIVERSITAT DE BARCELONA, (amb CIF Q0818001J i domicili a la Gran Via de les Corts Catalanes, 585 -08007 Barcelona) com a responsable del tractament de les dades personals, l'informa que pot contactar amb el Delegat de Protecció de Dades mitjançant escrit a l'adreça postal (Travessera de les Corts, 131-159, Pavelló Rosa, 08028 - Barcelona), o mitjançant un missatge de correu electrònic

  b) Vostè té dret a accedir a les seves dades, sol·licitar la rectificació de les dades inexactes o, si és el cas, sol·licitar-ne la supressió, així com limitar-ne el tractament, oposar-se i retirar el consentiment del seu ús per a determinades finalitats. Aquests drets els pot exercir mitjançant escrit a l'adreça postal o mitjançant un missatge de correu electrònic a l'adreça esmentada en el paràgraf

anterior. Així mateix, l'informem del seu dret a presentar una reclamació davant de l'Agència Catalana de Protecció de Dades en el cas de qualsevol actuació de la Universitat de Barcelona que consideri que vulnera els seus drets.

- En el supòsit de projectes de recerca i tesis de doctorat basats en enquestes, qüestionaris i semblants, per tal de complir amb la normativa de protecció de dades i garantir la privacitat dels voluntaris participants en la recerca i de les seves dades personals, convé utilitzar el programa FORMS disponible al núvol UB (https://www.ub.edu/portal/web/iub/nuvol-ub).
- Cal que afegiu el model del document d'informació i el de consentiment que s'utilitzarà, així com el procediment emprat per obtenir-lo.
  Al web de la CBUB hi ha un model orientatiu de consentiment informat:
  http://www.ub.edu/comissiobioetica/ca/model-orientatiu-full-consentiment-informat
- En el cas que el Projecte tingui previst d'emprar mostres biològiques d'origen humà dipositades en un Biobanc, o bé que formin part d'una col·lecció de mostres per a fins de recerca biomèdica conservada fora de l'àmbit organitzatiu d'un Biobanc, cal afegir una còpia del full de sol·licitud de cessió de les mostres a un investigador concret (citar-lo) i per a un Projecte concret (citar-lo)."

En tots els casos, també cal afegir-hi:

- Compromís signat per l'investigador/a principal del projecte i/o pel director/a de la Tesi de doctorat de que la confidencialitat de les dades que es puguin obtenir en el projecte serà escrupolosament observada. Si es considera que algun resultat del projecte pot afectar o pot ser d'interès per les persones o col·lectius participants, caldrà comunicar-los-hi.
- Compromís de no cedir ni utilitzar les mostres per a altres estudis diferents. En aquest darrer supòsit, els investigadors han de sol·licitar abans el corresponent informe favorable de la Comissió.
- Compromís de que l'investigador principal del Projecte o el Director de la Tesi de doctorat tindrà en compte els Comunicats de la Comissió sobre la participació d'estudiants en projectes de recerca (només en els casos que escaigui), sobre possibles problemes ètics en les publicacions científiques i sobre els fulls d'informació als voluntaris participants en projectes de recerca.
  Aquests Comunicats es poden trobar al web de la Comissió:
  http://www.ub.edu/recerca/comissiobioetica.htm
- Compromís que no es cometrà cap tipus de plagi en els continguts de la present tesi de doctorat, TFG o TFM, per part dels membres que hi puguin participar, atès el que diuen els Codis de Bones Pràctiques en recerca.
- Còpia escanejada de l' informe favorable del Comitè Ètic d'Experimentació Animal (CEEA) en cas que es realitzi també experimentació amb animals.
- Cal especificar les compensacions econòmiques o altres previstes en el Projecte, tant per a l'investigador principal (o per a l'equip investigador) com per a les persones o col·lectius que hi participin. En cas que no se'n prevegin, cal fer-ho constar així.
- Especificació de la cobertura de l'assegurança subscrita a nom dels voluntaris participants, si escau.
- Altres dades que es consideri importants.

Aquest full s'ha adjuntar a la sol·licitud del projecte de recerca que queda dipositada a l'Oficina de Gestió de la Recerca i Convenis.

UNIVERSITAT DE BARCELONA

COMISSIÓ DE BIOÈTICA

JUSTIFICACIÓ CIENTÍFICA I ÈTICA DEL PROJECTE D'INVESTIGACIÓ

Impacte i beneficis
En els darrers anys, els problemes de salut mental s'han incrementat entre els estudiants universitaris, especialment en les dones. La inactivitat física i el sedentarisme afecten greument la salut mental durant la vida universitària. És fonamental desenvolupar estratègies personalitzades que afavoreixin la salut cerebral i el benestar psicològic en els inicis de l'edat adulta i aprofundir en la comprensió dels mecanismes neurobiològics i conductuals subjacents a les noves intervencions relatives als estils de vida.

El principal objectiu del present projecte consisteix a comprendre els mecanismes neuro-psico-biològics i els efectes d'estratègies d'intervenció ment i cos en la població jove, amb i sense realitat virtual, amb la finalitat de promoure el desenvolupament d'estratègies personalitzades per millorar el seu funcionament cognitiu i el seu benestar emocional. Per això, es duran a terme dos estudis controlats aleatoritzats de mètode mixt. **El primer estudi** té com a objectiu avaluar l'eficàcia de l'entrenament funcional d'alta intensitat (HIFT), el Qigong (QG) i la reducció de l'estrès basada en l'atenció plena (MBSR) per afavorir la salut cerebral, el funcionament cognitiu i el benestar psicològic dels estudiants universitaris. A més, es pretén explorar el paper de variables socio-demogràfiques i personals (com el sexe), així com el de les molècules (microbiota), el cervell (estructures i funcions cerebrals), els components fisiològics (resultats relacionats amb l'estat físic, l'activitat física i la variabilitat del ritme cardíac) i psicològics (atenció plena, qualitat de vida, qualitat del son i fatiga) com a mediadors i moderadors en els canvis induïts per la intervenció. Per altra banda, tenint en compte que un entorn de realitat virtual (RV) immersiu i innovador pot oferir beneficis addicionals i millorar l'adherència a l'entrenament en aquesta població, **el segon estudi** consistirà a desenvolupar i explorar la viabilitat i l'eficàcia d'aquestes intervencions realitzades a través de realitat virtual (HIFT-VR, QG-VR, MBSR-VR), sobre el benestar cognitiu i psicològic dels estudiants universitaris. Les intervencions inclouran tres sessions setmanals d'una hora de durada durant un període de 12 setmanes. S'espera que les intervencions ment i cos proposades mostrin resultats diferencials en relació al funcionament cognitiu i al benestar psicològic, basant-se en els mecanismes neuro-psico-biològics subjacents. Els entorns de realitat virtual proporcionaran una major adherència i beneficis addicionals respecte als obtinguts a través de l'entrenament convencional. Els resultats derivats del present estudi permetran desenvolupar estratègies personalitzades per promoure la salut cerebral i el benestar psicològic en adults joves. Les intervencions per a la salut mental que inclouen la promoció de la salut cerebral per a un envelliment exitós han de començar aviat en el curs de la vida, i aquesta és una contribució principal d'aquest projecte a la nostra societat.

L'estudi 1 inclourà a 174 estudiants universitaris sans, entre 18 i 25 anys, que acceptin participar de l'estudi, i compleixin amb els criteris d'inclusió i exclusió establerts. Per a la difusió i el contacte amb els potencials participants universitaris, utilitzarem diversos mètodes: Penjarem cartells informatius a les diferents universitats de Barcelona, farem ús de plataformes de xarxes socials (WhatsApp, Facebook, X), recorrerem a la tècnica de reclutament "bola de neu", i comptarem amb la col·laboració de professors que informaran als seus estudiants. Els alumnes interessats podran posar-se en contacte amb nosaltres enviant un correu electrònic a una adreça específica del projecte o enviant un WhatsApp a un número de telèfon designat per aquesta recerca vegada rebem el seu contacte, ens posarem en comunicació amb ells per oferir-los tota la informació pertinent respecte a l'estudi. Els

participants seran assignats aleatòriament mitjançant una seqüència d'assignació generada per ordinador i estratificada per sexe a cada una de les tres intervencions. Als participants se'ls demanarà que completin un qüestionari inicial que inclourà informació demogràfica (edat; sexe: femení, masculí, intersexual, altres; i gènere: dona, home, no binari, altres; nacionalitat; relacions; àrea d'estudi; universitat; curs; rendiment acadèmic; situació laboral i estatus socioeconòmic) i antecedents de salut. Es realitzaran mesures cognitives, psicològiques i físiques a tots els participants en un període de dues setmanes abans de les intervencions, que es repetiran en un període de dues setmanes després de completar les intervencions. Hi haurà un seguiment 12 setmanes després de la finalització de l'estudi. Aquest seguiment avaluarà els qüestionaris en línia sobre el benestar físic i psicològic. En un subgrup seleccionat aleatòriament de 90 participants (30 de cada grup), també procedirem a l'obtenció de mostres biològiques (fecals) i imatges cerebrals (RM) abans i després de les intervencions. A més, al finalitzar el programa, se sol·licitarà a un subconjunt de 24 participants (8 de cada grup) que participin en una entrevista semiestructurada per conèixer les seves experiències i avaluar la significació clínica de possibles canvis.

L’estudi 2 comprendrà un total de 45 estudiants universitaris elegibles, d'edats compreses entre 18 i 25 anys, que seran assignats aleatòriament a tres grups: HIFT-VR, QG-VR i MBSR-VR (n=15). A més dels criteris generals d'exclusió, se'ls demanarà als participants si tenen alguna contraindicació coneguda o algun problema que pugui condicionar l'ús de la realitat virtual (com ara epilèpsia o marejos). Es realitzaran les mateixes avaluacions que en l'Estudi 1, excepte les RM i les mostres fecals.

La justificació ètica del projecte implica supervisió acurada de la informació detallada que es proporciona als participants i dels compromisos adquirits per l’equip investigador.

Possibles riscos i molèsties de les diferents proves i intervencions

Exploració Neuropsicològica
Es tracta d'una prova curta i totalment innòcua, amb una durada aproximada d'1 hora i mitja, en què es valoraran diferents funcions com la memòria, atenció, llenguatge, funcions executives i velocitat de processament de la informació, per tal d'obtenir un perfil detallat de l’estat cognitiu del participant. Aquestes proves seran administrades presencialment per l’equip de neuropsicòlegs. També s’administraran qüestionaris per a l’avaluació de l’estat emocional, estrès, son, i qualitat de vida, entre d’altres que es farà en format online. Aquesta exploració tindrà lloc dues vegades. Una al principi, durant el reclutament del participant, i una altra al finalitzar la intervenció, a les 12 setmanes. Hi haurà un seguiment 12 setmanes després de la finalització de l'estudi. Aquest seguiment avaluarà els qüestionaris en línia sobre el benestar físic i psicològic.

Exploració de la condició física i de l’estat físic
L’exploració de l’activitat física i de l’estat físic es realitzarà mitjançant dispositius de registre de l’activitat física i proves que impliquen un risc mínim pel participant. Aquesta avaluació tindrà una durada d’aproximadament 1 hora. En el cas que el participant manifesti alguna molèstia, dolor o problemes durant l’avaluació, tindrà la possibilitat de parlar-ho amb l’investigador principal o amb els investigadors mèdics que participen en l’estudi, per tal d’orientar-lo i descartar cap risc en la seva participació en l’estudi. Aquesta exploració tindrà lloc dues vegades. Una al principi, durant el reclutament del participant, i una altra al finalitzar la intervenció, a les 12 setmanes.

Estudi de ressonància magnètica

Les tècniques que s'aplicaran, la ressonància magnètica estructural, funcional, i de tensor de difusió, són tècniques no invasives. No existeix risc important per als participants donat que es tracta d'una prova que no utilitza radiacions ionitzants (a diferència de les radiografies) i fins ara, no s'han trobat efectes adversos. Algunes de les incomoditats amb les que es pot trobar el pacient són les següents: el estudis són més llargs (poden arribar a durar una hora) i requereixen la col·laboració del pacient (romandre molt quiet i col·laborar en la respiració). Existeix un perill inherent al camp magnètic del mateix equip donat que pot atraure objectes metàl·lics o espatllar instruments electrònics. Per tant, no és possible introduir participants que portin marcapàs, alguns implants o clips cerebrals. Per aquest motiu, als participants se li passarà un qüestionari en el full de consentiment informat en relació a aquests inconvenients tant per part de l'investigador responsable en el moment del reclutament com per part del tècnic de ressonància magnètica per tal d'assegurar-nos que el participant pot entrar a fer-se la prova sense cap risc per la seva salut. Donat que es desconeixen els efectes adversos del camp magnètic durant el primer trimestre d'embaràs, es prefereix no realitzar estudis en dones embarassades amb menys de 12 setmanes de gestació. La prova de ressonància magnètica tindrà lloc dues vegades en els participants corresponents: una en la inclusió del pacient en l'estudi (basal) i l'altra en acabar el tractament (a les 12 setmanes). L'estudi de ressonància magnètica es realitzarà al Centre de Diagnòstic per la Imatge de l'Hospital Clínic i Provincial de Barcelona (HCB). L'equip investigador recollirà directament en un disc dur encriptat les imatges de les proves de neuroimatge realitzades a l'HCB, pel posterior tractament de les mateixes per part de l'equip investigador.

Recollida de mostra de femta

Factors individuals (l'edat o el gènere), l'estil de vida (activitat física, la dieta, l'ús d'antibiòtics) i l'estrès o les malalties caracteritzen de manera diferenciada la població bacteriana de l'intestí humà, que forma la microbiota intestinal. L'estudi de les mostres de femta pre i post-intervenció, en els participants corresponents, permetrà la detecció dels canvis fisiològics associats a la realització dels diferents tipus d'intervenció i relacionar-los amb les altres variables de l'estudi. Es una prova no invasiva i de fàcil obtenció, provocant cap molèstia en el participant. Se li demanarà una mostra de femta per analitzar la microbiota, abans i un cop finalitzi la intervenció. La mostra la portarà el pacient des de casa seva i serà recollida pel personal investigador.

Intervencions

Les intervencions d'*Entrenament funcional d'alta intensitat* (HIFT), *QiGong* (QG) i *Mindfulness basada en la reducció de l'estrès* (MBSR) tindran una durada de 12 setmanes. Es realitzaran tres sessions setmanals, en format en línia en grups de 12-15 participants (Estudi 1) o realitat virtual individualment (Estudi 2). El protocol d'entrenament de la intervenció de HIFT es dissenyarà basant-se en entrenaments Cross-Training, posant èmfasi en l'entrenament d'interval d'alta intensitat combinat amb moviments funcionals d'entrenament de resistència. La intervenció de QG, , un dels exercicis tradicionals xinesos més populars, inclourà la seqüència Baduanjin, considerada com una de les formes més ancestrals i beneficioses per a la salut física i mental dins de la tradició del QiGong. La intervenció de *Mindfulness basada en la reducció de l'estrès* (MBSR), és una intervenció totalment innòcua. Consisteix en tècniques de meditació, relaxació i ioga, seguint el programa oficial de MBSR dissenyat per Kabat-Zinn. Aquestes intervencions no suposen cap risc per a la salut del participant. Tanmateix, en cas que el participant manifesti alguna molèstia, dolor o problemes durant la intervenció o transcurs de l'estudi, tindrà la possibilitat de parlar-ho

amb l'investigador principal i els investigadors mèdics de l'estudi, per tal de descartar cap risc en la seva participació en l'estudi.

Realitat virtual
Pel que fa a l'ús dels dispositius de realitat virtual, es prendran les mesures adequades per prevenir qualsevol risc. Els participants seran instruïts per ajustar adequadament les ulleres de realitat virtual. També se'ls informarà que s'han de treure les ulleres de realitat virtual durant 30 segons a la meitat de les intervencions. Tot i que, donada l'edat dels participants, no esperem dificultats amb els controladors i les funcions del dispositiu i l'entorn de realitat virtual, estarem atents a qualsevol dificultat. Prendrem precaució amb les instruccions i les preguntes per assegurar-nos que tot es desenvolupa sense problemes.

INFORMACIÓ DETALLADA QUE REBRÀ EL VOLUNTARI SOTMÈS A LES PROVES

Els participants seran informats de forma clara i detallada de les característiques i objectius d'aquest projecte i hauran de completar i signar els consentiments informats abans de continuar amb l'estudi.

Abans de la realització de cada una de les proves, cada participant rebrà informació detallada sobre l'objectiu i durada d'aquestes. Un cop obtinguts els resultats de les proves neuropsicològiques i de ressonància magnètica es facilitarà un informe escrit al propi interessat.

L'avaluació clínica, neuropsicològica i física serà realitzada en tots els casos per personal especialitzat en la Facultat de Psicologia de la Universitat de Barcelona. La RM s'efectuarà en el Centre de Diagnòstic per la Imatge de l'Hospital Clínic i Provincial de Barcelona.

La programació per a la realització de les diferents proves es farà mitjançant contacte telefònic amb el participant per part d'algun dels membres de l'equip investigador, per tal de fixar els dies i hores que siguin convenients pel participant.

El responsable del tractament de les dades personals és la Secretaria General de la Universitat de Barcelona (amb CIF Q0818001J i domicili a la Gran Via de les Corts Catalanes, 585 -08007 Barcelona).

Per assegurar la confidencialitat i seguretat, les mesures que es prendran són les següents:

- Emmagatzematge de dades: es farà ús de plataformes segures autoritzades per la Universitat de Barcelona (UB), incloent el Núvol OneDrive, Microsoft Forms, i Qualtrics.

- Protecció de la identitat dels participants: Les dades es tractaran de forma seudonimitzada. Això vol dir que, encara que les dades recollides estaran vinculades a un codi únic per a cada participant, la informació que permetria identificar directament als participants (com ara noms o adreces de correu electrònic) serà emmagatzemada de manera separada. A més, aquesta informació identificativa estarà restringida i només tindrà accés a ella la investigadora principal.

UNIVERSITAT DE BARCELONA

COMISSIÓ DE BIOÈTICA

COMPROMISOS ADQUIRITS PER L'EQUIP INVESTIGADOR

L'equip investigador es compromet, amb cadascun dels participants que participin en l'estudi a:

- Mantenir la confidencialitat de les dades obtingudes en el projecte d'investigació i garantir, en tot moment, que a partir de la primera entrevista serà identificat pel seu nom i per un codi numèric que mantindrà anònima la seva identitat per a la resta d'investigadors.
- Garantir que els participants puguin abandonar l'estudi en el moment en que així ho desitgin.
- Informar, de manera clara i senzilla, sobre els resultats de les proves neuropsicològiques i de ressonància magnètica que puguin ser d'interès pels participants.
- Utilitzar les dades obtingudes pel projecte d'investigació del qual s'ha informat al participant. Els resultats neuropsicològics, de microbiota intestinal, i de ressonància magnètica no seran utilitzats per a altres possibles projectes ni cedits a altres equips d'investigació, sense sol·licitar abans el corresponent informe de la Comissió de Bioètica de la Universitat de Barcelona.
- Tenir en compte els Comunicats de la Comissió sobre possibles problemes ètics en les publicacions científiques i sobre els fulls d'informació als voluntaris participants en projectes de recerca.
- Cada participant rebrà 30€ com a compensació econòmica.
- No es cometrà cap tipus de plagi en els continguts de la present investigació, per part dels membres que hi puguin participar, atès el que diuen els Codis de Bones Pràctiques en recerca.

CONSENTIMENT INFORMAT DELS SUBJECTES QUE PARTICIPEN EN ELS ESTUDIS

Cadascun dels subjectes que intervinguin en l'estudi 1 ó 2 signarà el full de consentiment informat corresponent que s'adjunta.

I, perquè així consti, l'investigador principal signa aquest document a Barcelona, a 20 de setembre de 2023.

Dra. Maria Mataró
Investigadora principal del projecte

UNIVERSITAT DE BARCELONA

COMISSIÓ DE BIOÈTICA

DOCUMENT INFORMATIU PER ALS PARTICIPANTS

Joves actius, ment sana: Estratègies per a la salut cerebral i el benestar psicològic en joves (YoungFitT: Young Fitness Technology). Estudi 1.

1. INTRODUCCIÓ I FINALITAT DE L'ESTUDI

El present estudi té com a objectiu principal investigar els efectes de diferents modalitats cos-ment, en concret del HIFT (*High-Intensity Functional Training*), Qigong i MBSR (*Mindfulness-Based Stress Reduction*) en la salut cerebral, la cognició i el benestar emocional. A més, s'exploraran els mecanismes biològics subjacents i es tindran en compte els factors que poden influir en les diferències individuals en la resposta del cervell jove a aquestes modalitats d'exercici. Es tracta d'un projecte en el qual participen de forma coordinada investigadors del Departament de Psicologia Clínica i Psicobiologia de la Facultat de Psicologia de la Universitat de Barcelona, la Facultat de Psicologia Blanquerna de la Universitat Ramon Llull, l'Institut Nacional d'Educació Física de Catalunya (INEFC) i l'Institut d'Investigació en Atenció Primària Jordi Gol.

En el context d'aquesta investigació li demanem la seva col·laboració en l'estudi, ja que compleix els criteris d'inclusió següents: tenir una edat entre 18 i 25 anys i estar cursant estudis universitaris. Aquesta col·laboració implica participar primer en una fase d'avaluació, després en una fase d'intervenció i en una fase d'avaluació posterior, que s'expliquen a continuació:

- Avaluació basal cognitiva, benestar emocional, estat físic, microbiota i neuroimatge.
1. *Recollida de dades sociodemogràfiques personals* (edat, sexe i gènere, …) i entorn de salut.
2. *Exploració Neuropsicològica*. Es tracta d'una prova curta, amb una durada aproximada d'1 hora i mitja, en què es valoraran diferents funcions com la memòria, atenció, llenguatge, funcions executives i velocitat de processament de la informació, per tal d'obtenir un perfil detallat del seu estat cognitiu. També s'administraran qüestionaris per a l'avaluació de l'estat emocional, estrès, son, i qualitat de vida, entre d'altres.
3. *Exploració de la condició física i de l'estat físic* a través de proves físiques amb una durada d'1 hora.
4. *Anàlisi de microbiota*. Aportació d'una petita mostra de femta per a les anàlisis de microbiota.
5. *Prova de Ressonància Magnètica Estructural i Funcional*. La ressonància magnètica no comporta cap risc per a la salut ja que és una tècnica completament segura, encara que li pot ocasionar alguna molèstia per soroll o incomoditat, com per exemple, claustrofòbia o angoixa durant la realització de la mateixa. Aquesta tècnica està contraindicada en alguns casos de portadors de pròtesis o altres metalls, per exemple, protesis cardíaques, d'oïda o marcapassos. En cas de que algú dels participants portés aquestes pròtesis s'avaluaria individualment. Totes les ressonàncies seran avaluades per un neurorradiòleg. En el cas d'alguna troballa clínicament significativa se'ls informarà i podran recollir l'informe i les imatges en el centre. L'objectiu d'aquesta prova és estudiar l'estat i funcionament de diferents àrees cerebrals, abans i després de les intervencions, fet que proporcionarà una visió profundament detallada de com les intervencions afecten el cervell i de quina manera aquests efectes es relacionen amb els beneficis cognitius i emocionals.
Tant la Ressonància Magnètica com l'anàlisi de la microbiota es duran a terme de manera aleatòria en una selecció de participants.

- Participació en l'estudi dels efectes de tres intervencions.
De forma aleatoritzada, podrà ser designat/da en un dels següents grups d'intervenció: *Mindfulness basada en la reducció de l'estrès* (MBSR), *Qigong* (QG) i *Entrenament funcional d'alta intensitat Functional Training* (HIFT). Les intervencions tindran una durada de 12 setmanes, es realitzaran en format en línia, en grups de 12-15 participants. Les intervencions HIFT i QG inclouran tres sessions per setmana: dos grupals de 60 minuts en línia guiades per experts i una sessió individual autònoma. La intervenció de MBSR seguirà el programa oficial de MBSR dissenyat per Kabat-Zinn amb algunes

adaptacions durant tres dies a la setmana, inclosa una sessió sincrònica en línia de 120 minuts i dues sessions de 20 minuts. A l'inici de les intervencions, rebran una sessió informativa i un manual escrit amb mesures de seguretat per evitar lesions o altres possibles experiències adverses.

- Avaluació posterior a la intervenció.
L'avaluació cognitiva, del benestar emocional, de l'estat físic es durà també a terme després de les intervencions i als participants corresponents se'ls farà l'anàlisi de la microbiota, la Ressonància Magnètica. Hi haurà un seguiment 12 setmanes després de la finalització de l'estudi. Aquest seguiment avaluarà els qüestionaris en línia sobre el benestar físic i psicològic. Al final del programa, es demanarà a un subconjunt de participants que participin en una entrevista qualitativa.

2. RISCOS I BENEFICIS DE L'ESTUDI. La participació en aquest estudi no suposa cap risc per a la salut, ja que les exploracions que es realitzaran són completament innòcues. En canvi, suposa importants beneficis per a vostè i per a la societat, ja que estan dirigides a determinar els efectes d'aquestes intervencions en la salut cerebral, en el seu estat cognitiu, emocional i de salut, i investigar els mecanismes i factors implicats.

3. RETIRADA DE L'ESTUDI. La seva participació és completament voluntària, té total llibertat per negar-se a participar en l'estudi o per suspendre la seva col·laboració quan ho desitgi, sense que aquesta decisió pugui ocasionar-li cap perjudici.

4. CONFIDENCIALITAT I PROTECCIÓ DE DADES. Només l'investigador principal tindrà accés a la teva identitat. La Secretaria General de la Universitat de Barcelona és, en relació amb el compliment de la normativa de protecció de dades personals, la responsable legal del tractament d'aquestes dades en el marc del projecte. Això no implica que tingui accés ni a la teva identitat, dades que es generen, ni al formulari de consentiment informat, excepte en cas d'obligació legal (per exemple, si algú presenta una reclamació davant l'autoritat de control de protecció de dades o davant d'un jutge o tribunal). D'acord amb el Reglament general de protecció de dades personals de la Unió Europea, t'informem que les dades de contacte de la Secretaria General són Gran Via de les Corts Catalanes, 585, 08007 Barcelona i , per si en algun moment vols exercir els drets que et reconeix la normativa de protecció de dades personals (pots accedir a les teves dades i sol·licitar-ne la rectificació, supressió, oposició, portabilitat o limitació). En cas que vulguis exercir-los, hauràs d'adjuntar una fotocòpia del DNI o d'altre document vàlid que t'identifiqui. Les dades personals que es recullin únicament s'utilitzaran amb la finalitat de gestionar i executar el projecte de recerca YoungfitT segons el teu consentiment, que pots revocar en qualsevol moment sense que tingui efectes retroactius. Els destinataris de les dades personals són la mateixa Universitat i, en concret, l'equip de recerca del projecte i, si n'hi ha, els encarregats del tractament de les dades. No se cedeixen dades a tercers, llevat que sigui per obligació legal. Aquestes dades personals es conservaran fins que s'hagin assolit els objectius del projecte i se n'hagin publicat els resultats (aproximadament, fins a 5 anys des de la finalització del projecte). Si consideres que els teus drets no s'han atès adequadament, pots comunicar-ho al delegat de protecció de dades de la UB per correu postal (Gran Via de les Corts Catalanes, 585, 08007 Barcelona) o per correu electrònic . També pots presentar una reclamació davant l'Autoritat Catalana de Protecció de Dades (https://apdcat.gencat.cat/ca/inici). Aquest estudi respecta el "Reglament (UE) 2016/679 del Parlament i del Consell, de 27 d'abril de 2016, relatiu a la protecció de les persones físiques pel que fa al tractament de dades personals i a la lliure circulació d'aquestes dades i pel qual es deroga la Directiva 95/46/CE (Reglament general de protecció de dades)" i per l'altra la "Llei orgànica 3/2018, de 5 de desembre, de protecció de dades personals i garantia dels drets digitals".

5. COMPENSACIONS. La participació en l'estudi tindrà una compensació econòmica de 30€.

6. DRET A CONÈIXER ELS RESULTATS. Al final de l'estudi, si ho desitja, podrà conèixer els resultats de la seva participació i de l'estudi.

7. SIGNATURA EN DOS EXEMPLARS. El document de participació en l'estudi es signarà en dos exemplars idèntics i una còpia impresa serà per a la persona participant.

8. PREGUNTES I CONSULTES. Els participants tenen dret a fer totes les preguntes que els hi semblin pertinents respecte de les característiques de l'estudi i de la seva pròpia participació en aquest. Per a qualsevol dubte o aclariment referent a l'estudi, pot dirigir-se a l'equip responsable. A continuació li detallem la informació de contacte: Professional responsable de l'estudi: Dra. Maria Mataró. Catedràtica de Psicobiologia. Universitat de Barcelona.

Telèfons de contacte: Telèfon associat al projecte; Laboratori de Neuropsicologia Facultat Psicologia. Universitat de Barcelona. Correu electrònic:

a) De conformitat amb l'establert a l'esmentada regulació, la UNIVERSITAT DE BARCELONA, (amb CIF Q0818001J i domicili a la Gran Via de les Corts Catalanes, 585 -08007 Barcelona) com a responsable del tractament de les dades personals, l'informa que pot contactar amb el Delegat de Protecció de Dades mitjançant escrit a l'adreça postal (Travessera de les Corts, 131-159, Pavelló Rosa, 08028 - Barcelona), o mitjançant un missatge de correu electrònic

b) Vostè té dret a accedir a les seves dades, sol·licitar la rectificació de les dades inexactes o, si és el cas, sol·licitar-ne la supressió, així com limitar-ne el tractament, oposar-se i retirar el consentiment del seu ús per a determinades finalitats. Aquests drets els pot exercir mitjançant escrit a l'adreça postal o mitjançant un missatge de correu electrònic a l'adreça esmentada en el paràgraf anterior. Així mateix, l'informem del seu dret a presentar una reclamació davant de l'Agència Catalana de Protecció de Dades en el cas de qualsevol actuació de la Universitat de Barcelona que consideri que vulnera els seus drets.

UNIVERSITAT DE BARCELONA

COMISSIÓ DE BIOÈTICA

CONSENTIMENT INFORMAT DEL PARTICIPANT

Títol del projecte de recerca: Joves actius, ment sana: Estratègies per a la salut cerebral i el benestar psicològic en joves (YoungFitT: Young Fitness Technology). Estudi 1.

El voluntari ha de llegir i contestar les preguntes següents amb atenció: (Cal encerclar la resposta que es consideri correcta)

Ha llegit tota informació que li ha estat facilitada sobre aquest projecte?	SI / NO
Ha tingut l'oportunitat de preguntar i comentar qüestions sobre el projecte?	SI / NO
Ha rebut suficient informació sobre aquest projecte?	SI / NO
Ha rebut respostes satisfactòries a totes les preguntes?	SI / NO
Quin investigador li ha parlat d'aquest projecte? (nom i cognoms): …………………………………………	
Ha comprès que vostè és lliure d'abandonar aquest projecte sense que aquesta decisió pugui ocasionar-li cap perjudici?	SI / NO
En qualsevol moment	SI / NO
Sense donar-ne cap raó	SI / NO
Ha comprès els possibles riscos associats a la seva participació en aquest projecte?	SI / NO
Està d'acord en participar-hi?	SI / NO
Accepta participar en la prova de Ressonància Magnètica i l'anàlisi de la microbiota en cas de ser seleccionat?	SI / NO
Entenc que se'm pot sol·licitar participar en l'entrevista qualitativa.	SI / NO
Rebrà algun tipus de compensació per participar-hi?	SI / NO
Consent que les seves dades personals siguin tractades d'acord amb l'indicat?	SI / NO

Signatura: .... Data: ………………………

Nom i cognoms del voluntari: ....

En cas que més endavant vostè vulgui fer alguna pregunta o comentari sobre aquest projecte, o bé si vol revocar la seva participació en el mateix, si us plau contacti amb: Dra. Maria Mataró. Catedràtica de Psicobiologia. Dept. Psicologia clínica i Psicobiologia. Facultat de Psicologia. Pg. de la Vall d'Hebron 171. 08031 Barcelona. E-mail de contacte: ; Telèfons de contacte:

Lloc, data i signatura de l'investigador: ....

Exemplar per al participant / Exemplar per a l'investigador

UNIVERSITAT DE BARCELONA

COMISSIÓ DE BIOÈTICA

a) De conformitat amb l'establert a l'esmentada regulació, la UNIVERSITAT DE BARCELONA, (amb CIF Q0818001J i domicili a la Gran Via de les Corts Catalanes, 585 -08007 Barcelona) com a responsable del tractament de les dades personals, l'informa que pot contactar amb el Delegat de Protecció de Dades mitjançant escrit a l'adreça postal (Travessera de les Corts, 131-159, Pavelló Rosa, 08028 - Barcelona), o mitjançant un missatge de correu electrònic

b) Vostè té dret a accedir a les seves dades, sol·licitar la rectificació de les dades inexactes o, si és el cas, sol·licitar-ne la supressió, així com limitar-ne el tractament, oposar-se i retirar el consentiment del seu ús per a determinades finalitats. Aquests drets els pot exercir mitjançant escrit a l'adreça postal o mitjançant un missatge de correu electrònic a l'adreça esmentada en el paràgraf anterior. Així mateix, l'informem del seu dret a presentar una reclamació davant de l'Agència Catalana de Protecció de Dades en el cas de qualsevol actuació de la Universitat de Barcelona que consideri que vulnera els seus drets.

UNIVERSITAT DE BARCELONA

COMISSIÓ DE BIOÈTICA

DOCUMENT INFORMATIU PER ALS PARTICIPANTS

Joves actius, ment sana: Estratègies per a la salut cerebral i el benestar psicològic en joves amb realitat virtual (YoungFitT: Young Fitness Technology). Estudi 2.

1. INTRODUCCIÓ I FINALITAT DE L'ESTUDI

El present estudi se centra en investigar els efectes de diferents modalitats cos-ment realitzades en un entorn de realitat virtual (VR), específicament HIFT-VR (*High-Intensity Functional Training*), QG-VR (*Qigong*) i MBSR-VR (*Mindfulness-Based Stress Reduction*), sobre la salut cerebral, la cognició i el benestar emocional en estudiants universitaris. A més, s'exploraran els mecanismes biològics subjacents i es tindran en compte els factors que poden influir en les diferències individuals en la resposta del cervell jove a aquestes modalitats d'exercici. Es tracta d'un projecte en el qual participen de forma coordinada investigadors del Departament de Psicologia Clínica i Psicobiologia de la Facultat de Psicologia de la Universitat de Barcelona, la Facultat de Psicologia Blanquerna de la Universitat Ramon Llull, l'Institut Nacional d'Educació Física de Catalunya (INEFC) i l'Institut d'Investigació en Atenció Primària Jordi Gol.

En el context d'aquesta investigació li demanem la seva col·laboració en l'estudi, ja que compleix els criteris d'inclusió següents: tenir una edat entre 18 i 25 anys i estar cursant estudis universitaris.

Aquesta col·laboració implica participar primer en una fase d'avaluació, després en una fase d'intervenció i en una fase d'avaluació posterior, que s'expliquen a continuació:

- Avaluació basal cognitiva, benestar emocional, estat físic

1. *Recollida de dades sociodemogràfiques personals* (edat, sexe i gènere, …) i entorn de salut.
2. *Exploració Neuropsicològica*. Es tracta d'una prova curta, amb una durada aproximada d'1 hora i mitja, en què es valoraran diferents funcions com la memòria, atenció, llenguatge, funcions executives i velocitat de processament de la informació, per tal d'obtenir un perfil detallat del seu estat cognitiu. També s'administraran qüestionaris per a l'avaluació de l'estat emocional, estrès, son, i qualitat de vida, entre d'altres.
3. *Exploració de la condició física i de l'estat físic* a través de proves físiques amb una durada d'1 hora.

- Participació en l'estudi dels efectes de tres intervencions.

De forma aleatoritzada, podrà ser designat/da en un dels següents grups d'intervenció: *Mindfulness basada en la reducció de l'estrès* (MBSR-VR), *Qigong* (QG-VR) i *Entrenament funcional d'alta intensitat Functional Training* (HIFT-VR). Les intervencions tindran una durada de 12 setmanes, es realitzaran a través de la realitat virtual de forma autònoma. Les intervencions HIFT-VR i QG-VR inclouran tres sessions d'una hora cadascuna per setmana. La intervenció de MBSR seguirà el programa oficial de MBSR dissenyat per Kabat-Zinn amb algunes adaptacions durant també tres dies a la setmana.

Els participants d'aquests grups faran els programes de formació mentre miren les ulleres de realitat virtual. A l'inici de les intervencions, rebran una sessió informativa i un manual escrit amb mesures de seguretat per evitar lesions o altres possibles experiències adverses.

- Avaluació posterior a la intervenció. L'avaluació cognitiva, del benestar emocional, de l'estat físic també es durà a terme després de les intervencions. Hi haurà un seguiment 12 setmanes després de la finalització de l'estudi. Aquest seguiment avaluarà els qüestionaris en línia sobre el benestar físic i psicològic. Al final del programa, es demanarà a un subconjunt de participants que participin en una entrevista qualitativa.

2. RISCOS I BENEFICIS DE L'ESTUDI. La participació en aquest estudi no suposa cap risc per a la salut, ja que les exploracions que es realitzaran són completament innòcues. En canvi, suposa importants beneficis per a vostè i per a la societat, ja que estan dirigides a determinar els efectes en la salut cerebral, en el seu estat cognitiu, emocional i de salut, i investigar els factors implicats.

3. RETIRADA DE L'ESTUDI. La seva participació és completament voluntària, té total llibertat per negar-se a participar en l'estudi o per suspendre la seva col·laboració quan ho desitgi, sense que aquesta decisió pugui ocasionar-li cap perjudici.

4. CONFIDENCIALITAT I PROTECCIÓ DE DADES. Només l'investigador principal tindrà accés a la teva identitat. La Secretaria General de la Universitat de Barcelona és, en relació amb el compliment de la normativa de protecció de dades personals, la responsable legal del tractament d'aquestes dades en el marc del projecte. Això no implica que tingui accés ni a la teva identitat, dades que es generen, ni al formulari de consentiment informat, excepte en cas d'obligació legal (per exemple, si algú presenta una reclamació davant l'autoritat de control de protecció de dades o davant d'un jutge o tribunal). D'acord amb el Reglament general de protecció de dades personals de la Unió Europea, t'informem que les dades de contacte de la Secretaria General són Gran Via de les Corts Catalanes, 585, 08007 Barcelona i , per si en algun moment vols exercir els drets que et reconeix la normativa de protecció de dades personals (pots accedir a les teves dades i sol·licitar-ne la rectificació, supressió, oposició, portabilitat o limitació). En cas que vulguis exercir-los, hauràs d'adjuntar una fotocòpia del DNI o d'altre document vàlid que t'identifiqui. Les dades personals que es recullin únicament s'utilitzaran amb la finalitat de gestionar i executar el projecte de recerca YoungfitT segons el teu consentiment, que pots revocar en qualsevol moment sense que tingui efectes retroactius. Els destinataris de les dades personals són la mateixa Universitat i, en concret, l'equip de recerca del projecte i, si n'hi ha, els encarregats del tractament de les dades. No se cedeixen dades a tercers, llevat que sigui per obligació legal. Aquestes dades personals es conservaran fins que s'hagin assolit els objectius del projecte i se n'hagin publicat els resultats (aproximadament, fins a 5 anys des de la finalització del projecte). Si consideres que els teus drets no s'han atès adequadament, pots comunicar-ho al delegat de protecció de dades de la UB per correu postal (Gran Via de les Corts Catalanes, 585, 08007 Barcelona) o per correu electrònic . També pots presentar una reclamació davant l'Autoritat Catalana de Protecció de Dades (https://apdcat.gencat.cat/ca/inici). Aquest estudi respecta el "Reglament (UE) 2016/679 del Parlament i del Consell, de 27 d'abril de 2016, relatiu a la protecció de les persones físiques pel que fa al tractament de dades personals i a la lliure circulació d'aquestes dades i pel qual es deroga la Directiva 95/46/CE (Reglament general de protecció de dades)" i per l'altra la "Llei orgànica 3/2018, de 5 de desembre, de protecció de dades personals i garantia dels drets digitals".

5. COMPENSACIONS. La participació en l'estudi tindrà una compensació econòmica de 30€.

6. DRET A CONÈIXER ELS RESULTATS. Al final de l'estudi, si ho desitja, podrà conèixer els resultats de la seva participació i de l'estudi.

7. SIGNATURA EN DOS EXEMPLARS. El document de participació en l'estudi es signarà en dos exemplars idèntics i una còpia impresa serà per a la persona participant.

8. PREGUNTES I [illegible]ts tenen dret a fer totes les preguntes que els hi semblin pertinents respecte de les característiques de l'estudi i de la seva pròpia participació en aquest. Per a qualsevol dubte o aclariment referent a l'estudi, pot dirigir-se a l'equip responsable. A continuació li detallem la informació de contacte: Professional responsable de l'estudi:

Dra. Maria Mataró. Catedràtica de Psicobiologia. Universitat de Barcelona.

Telèfons de contacte: Telèfon associat al projecte; Laboratori de Neuropsicologia Facultat Psicologia. Universitat de Barcelona. Correu electrònic:

a) De conformitat amb l'establert a l'esmentada regulació, la UNIVERSITAT DE BARCELONA, (amb CIF Q0818001J i domicili a la Gran Via de les Corts Catalanes, 585 -08007 Barcelona) com a responsable del tractament de les dades personals, l'informa que pot contactar amb el Delegat de Protecció de Dades mitjançant escrit a l'adreça postal (Travessera de les Corts, 131-159, Pavelló Rosa, 08028 - Barcelona), o mitjançant un missatge de correu electrònic

b) Vostè té dret a accedir a les seves dades, sol·licitar la rectificació de les dades inexactes o, si és el cas, sol·licitar-ne la supressió, així com limitar-ne el tractament, oposar-se i retirar el consentiment del seu ús per a determinades finalitats. Aquests drets els pot exercir mitjançant escrit a l'adreça postal o mitjançant un missatge de correu electrònic a l'adreça esmentada en el paràgraf anterior. Així mateix, l'informem del seu dret a presentar una reclamació davant de l'Agència Catalana de Protecció de Dades en el cas de qualsevol actuació de la Universitat de Barcelona que consideri que vulnera els seus drets.

UNIVERSITAT DE BARCELONA

COMISSIÓ DE BIOÈTICA

CONSENTIMENT INFORMAT DEL PARTICIPANT

Títol del projecte de recerca: Joves actius, ment sana: Estratègies per a la salut cerebral i el benestar psicològic en joves amb realitat virtual (YoungFitT: Young Fitness Technology). Estudi 2.

El voluntari ha de llegir i contestar les preguntes següents amb atenció: (Cal encerclar la resposta que es consideri correcta)

Ha llegit tota informació que li ha estat facilitada sobre aquest projecte?	SI / NO
Ha tingut l'oportunitat de preguntar i comentar qüestions sobre el projecte?	SI / NO
Ha rebut suficient informació sobre aquest projecte?	SI / NO
Ha rebut respostes satisfactòries a totes les preguntes?	SI / NO
Quin investigador li ha parlat d'aquest projecte? (nom i cognoms):	
Ha comprès que vostè és lliure d'abandonar aquest projecte sense que aquesta decisió pugui ocasionar-li cap perjudici?	SI / NO
En qualsevol moment	SI / NO
Sense donar-ne cap raó	SI / NO
Ha comprès els possibles riscos associats a la seva participació en aquest projecte?	SI / NO
Està d'acord en participar-hi?	SI / NO
Entenc que se'm pot sol·licitar participar en l'entrevista qualitativa	SI / NO
Rebrà algun tipus de compensació per participar-hi?	SI / NO
Consent que les seves dades personals siguin tractades d'acord amb l'indicat?	SI / NO

Signatura: .... Data: ………………………

Nom i cognoms del voluntari: ....

En cas que més endavant vostè vulgui fer alguna pregunta o comentari sobre aquest projecte, o bé si vol revocar la seva participació en el mateix, si us plau contacti amb: Dra. Maria Mataró. Catedràtica de Psicobiologia. Dept. Psicologia clínica i Psicobiologia. Facultat de Psicologia. Pg. de la Vall d'Hebron 171. 08031 Barcelona. E-mail de contacte: Telèfons de contacte:

Lloc, data i signatura de l'investigador: ....

Exemplar per al participant / Exemplar per a l'investigador

a) De conformitat amb l'establert a l'esmentada regulació, la UNIVERSITAT DE BARCELONA, (amb CIF Q0818001J i domicili a la Gran Via de les Corts Catalanes, 585 -08007 Barcelona) com a responsable del tractament de les dades personals, l'informa que pot contactar amb el Delegat de Protecció de Dades mitjançant escrit a l'adreça postal (Travessera de les Corts, 131-159, Pavelló Rosa, 08028 - Barcelona), o mitjançant un missatge de correu electrònic

b) Vostè té dret a accedir a les seves dades, sol·licitar la rectificació de les dades inexactes o, si és el cas, sol·licitar-ne la supressió, així com limitar-ne el tractament, oposar-se i retirar el consentiment del seu ús per a determinades finalitats. Aquests drets els pot exercir mitjançant escrit a l'adreça postal o mitjançant un missatge de correu electrònic a l'adreça esmentada en el paràgraf anterior. Així mateix, l'informem del seu dret a presentar una reclamació davant de l'Agència Catalana de Protecció de Dades en el cas de qualsevol actuació de la Universitat de Barcelona que consideri que vulnera els seus drets.
