# Supplementary material for: The YoungFitT project: Study protocol for a randomized mixed-methods trial of physical exercise and mind-body interventions, with or without virtual reality, in university students
Source: PLoS One. 2025 Aug 1;20(8):e0328538. doi: 10.1371/journal.pone.0328538 (PMC12316210; doi:10.1371/journal.pone.0328538)
Supplement: S3 File — (PDF) [file pone.0328538.s003.pdf]

UNIVERSITY OF BARCELONA

BIOETHICS COMMISSION

FORM 1
Declaration of Human Experimentation or Use of Human-Origin Biological Samples

☒ I, the undersigned, principal investigator of the project entitled:

"Active Youth, Healthy Mind: Strategies for Brain Health and Psychological Well-being in Young People: A Randomized Mixed-Methods Study With and Without Virtual Reality (YoungFitT: Young Fitness Technology),"

declare that:

☒ Yes, the project involves experimentation with human beings
☒ Yes, biological samples of human origin will be used

Full Name: Maria Mataró Serrat

Department / Unit: Department of Clinical Psychology and Psychobiology / Psychobiology Section

Postal Address: Pg. Vall d'Hebron, 171

Signature and Date:

September 20, 2023

UNIVERSITY OF BARCELONA

BIOETHICS COMMISSION

Documents to be Attached:

In all cases: A brief scientific and ethical justification of the research project, including a description of the expected impact on participants and anticipated benefits and risks resulting from the research.

A) If there is already a **favorable** opinion from the Ethics Committee of the center where the experimentation will be conducted or from which samples will be provided, a scanned copy must be attached.

B) In other cases, the following must be attached:

- Detailed information that will be provided to the volunteer undergoing the procedures. In cases involving the participation of minors in research projects or doctoral theses, the consent form must include not only the signature of the parents or legal guardians but also explicit assent from the minors themselves, who must have been previously informed—in accordance with their level of comprehension—about the nature, procedures, and aims of the project. The research team must ensure that the minor’s well-being and comfort are respected throughout the entire experimental process, with special attention to any refusal or reluctance to continue participation.

Data Protection Regarding Personal Information

- According to Regulation (EU) 2016/679 of 27 April of the European Union regarding the protection of natural persons in relation to the processing of their personal data, and Organic Law 3/2018 of 5 December on the protection of personal data and the guarantee of digital rights, in cases where personal data (including biometric, genetic, or other personal data, especially involving children) are obtained during the execution of the research project or doctoral thesis, it must be explicitly stated: Who is responsible for processing and storing the collected data; Where the data will be stored and/or processed; What type of data is being collected, specifying whether it is coded, pseudonymized, or anonymized.
- Moreover, both senior researchers and doctoral students must include the following paragraphs in the information and consent forms provided to participants:

a) In accordance with the aforementioned regulations, the UNIVERSITY OF BARCELONA (with tax ID CIF Q0818001J and address at Gran Via de les Corts Catalanes, 585 - 08007 Barcelona), as the entity responsible for processing personal data, informs you that you may contact the Data Protection Officer in writing at Travessera de les Corts, 131-159, Pavelló Rosa, 08028 - Barcelona, or by email

b) You have the right to access your data, request correction of inaccurate data, or, if applicable, request deletion, restrict processing, object to processing, and withdraw your consent for specific purposes. These rights can be exercised in writing at the address or via the email mentioned above. Additionally, you are informed of your right to file a complaint with the Catalan Data Protection Authority if you believe that any actions by the University of Barcelona violate your rights.

- For research projects and doctoral theses based on surveys, questionnaires, and similar tools, to comply with data protection regulations and ensure participants' privacy, it is recommended to use the FORMS program available on UB's cloud platform (https://www.ub.edu/portal/web/iub/nuvol-ub).
- The model of the information and consent document that will be used, as well as the procedure for obtaining it, must be included. A template for informed consent can be found on the CBUB website:

http://www.ub.edu/comissiobioetica/ca/model-orientatiu-full-consentiment-informat

- If the project plans to use biological samples of human origin deposited in a biobank, or part of a sample collection for biomedical research purposes held outside the organizational scope of a biobank, a copy of the sample transfer request form must be provided. This must indicate the specific investigator and project.

In all cases, the following must also be attached:

- A signed commitment from the principal investigator and/or the doctoral thesis supervisor stating that the confidentiality of the data obtained during the project will be strictly maintained.
- If any findings from the project could affect or be of interest to participants or their communities, they must be informed.
- A commitment not to transfer or use the samples for other studies. For any such intention, the corresponding favorable report from the Bioethics Commission must be requested beforehand.
- A commitment that the principal investigator or doctoral thesis supervisor will adhere to the Commission's communications regarding student participation in research projects, ethical issues in scientific publications, and information sheets for volunteers in research projects. These communications can be found on the Commission's website:

http://www.ub.edu/recerca/comissiobioetica.htm

- A commitment that no form of plagiarism will occur in the doctoral thesis, TFG, or TFM by any involved parties, in accordance with the Codes of Good Research Practices.
- A scanned copy of the favorable report from the Animal Experimentation Ethics Committee (CEEA) must be included if animal experimentation is also conducted.

- Specification of any financial or other types of compensation foreseen in the project, for both the principal investigator (or research team) and the participants. If none are foreseen, this must be explicitly stated.
- Specification of the insurance coverage provided for participants, if applicable.
- Any other information deemed important.

This form must be attached to the research project request filed at the Office for Research and Agreements Management.

Scientific and Ethical Justification of the Research Project

Impact and Benefits
In recent years, mental health issues have increased among university students, particularly among women. Physical inactivity and sedentary behavior severely affect mental health during university life. It is essential to develop personalized strategies that promote brain health and psychological well-being in early adulthood and to further understand the neurobiological and behavioral mechanisms underlying new lifestyle interventions.

The main objective of this project is to understand the neuro-psycho-biological mechanisms and effects of mind-body intervention strategies in young people, with and without virtual reality, in order to develop personalized strategies to enhance cognitive function and emotional well-being. For this purpose, two randomized controlled mixed-methods studies will be conducted. Study 1 aims to evaluate the efficacy of High-Intensity Functional Training (HIFT), Qigong (QG), and Mindfulness-Based Stress Reduction (MBSR) in promoting brain health, cognitive function, and psychological well-being in university students. It also seeks to explore the role of sociodemographic and personal variables (such as sex), as well as biological markers (microbiota), brain structure and function, physiological components (e.g., physical fitness, physical activity, and heart rate variability), and psychological factors (e.g., mindfulness, quality of life, sleep quality, and fatigue) as potential mediators and moderators of intervention-induced changes. Furthermore, given that an immersive and innovative Virtual Reality (VR) environment may offer additional benefits and improve adherence in this population, Study 2 will develop and explore the feasibility and efficacy of delivering these interventions through VR (HIFT-VR, QG-VR, MBSR-VR) in enhancing cognitive and psychological well-being among university students. Interventions will consist of three one-hour weekly sessions over a 12-week period. It is expected that the proposed mind-body interventions will yield differential outcomes in cognitive function and psychological well-being, based on the underlying neuro-psycho-biological mechanisms. VR environments are anticipated to enhance adherence and offer added benefits compared to conventional training. Results from this study will contribute to the development of personalized strategies to promote brain health and psychological well-being in young adults. Mental health interventions that promote brain health for successful aging should begin early in life, and this is one of the project's main societal contributions.

Study 1 will include 174 healthy university students between 18 and 25 years old who agree to participate and meet the established inclusion and exclusion criteria. To recruit and reach potential participants, several methods will be used: posters at different universities in Barcelona, social media platforms (WhatsApp, Facebook, X), the snowball sampling technique, and collaboration with professors who will inform their students. Interested students can contact the research team by emailing the project-specific address or by sending a WhatsApp message to a designated number . Once contact is made, the team will provide all relevant information about the study. Participants will be randomly assigned using a computer-generated sequence stratified by sex into one of the three intervention groups. An initial questionnaire will be completed by participants, including demographic data (age, sex: female, male, intersex, other; gender: woman, man, non-binary, other; nationality; relationships; field of study; university; academic year; academic performance; employment status; and socioeconomic status) and health history. Cognitive, psychological, and physical assessments will be conducted within two weeks before the interventions and repeated within two weeks after completing the interventions. A follow-up will be conducted 12 weeks post-intervention via online questionnaires assessing physical and psychological well-being. In a randomly selected subgroup of 90 participants (30 per group), biological samples (fecal) and brain imaging (MRI) will be collected before and after the interventions. In addition, at the end of the program, a subset of 24 participants (8 per group) will participate in a semi-structured interview to share their experiences and assess the clinical significance of any observed changes.

Study 2 will include a total of 45 eligible university students aged 18 to 25, who will be randomly assigned into three groups: HIFT-VR, QG-VR, and MBSR-VR (n=15 per group). In addition to the general exclusion criteria, participants will be asked whether they have any known contraindications or conditions that may affect the use of virtual reality (e.g., epilepsy or dizziness). The same assessments as in Study 1 will be carried out, except for MRI and fecal samples, which will not be included in this study.

The ethical justification of the project involves careful supervision of the detailed information provided to participants and of the commitments made by the research team.

Potential Risks and Discomforts of the Various Tests and Interventions

Neuropsychological Assessment

This is a short and completely harmless test lasting approximately 1.5 hours. It evaluates various functions such as memory, attention, language, executive functions, and information processing speed, to obtain a detailed cognitive profile of the participant. These assessments will be conducted in person by the neuropsychology team. Online questionnaires will also be administered to assess emotional state, stress, sleep, and quality of life. These assessments will take place twice: at the beginning during participant recruitment, and at the end of the 12-week intervention period. A 12-week follow-up will assess online questionnaires regarding physical and psychological well-being.

Physical Condition and Fitness Assessment
Assessment of physical activity and fitness will be performed using physical activity monitoring devices and tests that involve minimal risk to participants. This evaluation will last approximately one hour. If any participant experiences discomfort, pain, or problems during the assessment, they will have the opportunity to discuss it with the principal investigator or the study's medical researchers, who will provide guidance and assess any risks involved. This assessment will also take place twice: at baseline (during recruitment) and after the 12-week intervention.

Magnetic Resonance Imaging (MRI)
The MRI techniques applied—structural, functional, and diffusion tensor imaging—are non-invasive and do not involve significant risk, as they do not use ionizing radiation (unlike X-rays). No adverse effects have been identified to date. Some potential inconveniences for participants include the longer duration (up to one hour) and the need to remain very still and follow breathing instructions. The magnetic field of the MRI device can attract metal objects or damage electronic devices. Therefore, participants with pacemakers, certain implants, or brain clips cannot be scanned. A questionnaire included in the informed consent form will screen for these issues, administered by both the responsible investigator during recruitment and the MRI technician. Because the effects of magnetic fields during the first trimester of pregnancy are unknown, the study will not include pregnant women under 12 weeks of gestation. MRI scans will be conducted twice for the relevant participants: at baseline and after 12 weeks of treatment. These scans will be performed at the Diagnostic Imaging Center of Hospital Clínic and Provincial de Barcelona (HCB). The research team will directly collect the neuroimaging data using an encrypted hard drive for subsequent analysis.

Fecal Sample Collection
Individual factors (such as age and gender), lifestyle (physical activity, diet, antibiotic use), and stress or illness affect the composition of the human gut microbiota. Analyzing pre- and post-intervention fecal samples will allow the detection of physiological changes associated with different types of interventions and their relationship with other study variables. This is a non-invasive and easy-to-obtain sample that causes no discomfort to participants. They will be asked to provide a fecal sample before and after the intervention. Participants will collect the sample at home and deliver it to the research staff.

Interventions
The interventions—High-Intensity Functional Training (HIFT), Qigong (QG), and Mindfulness-Based Stress Reduction (MBSR)—will each last for 12 weeks, with three weekly sessions. In Study 1, these will be delivered online in group formats of 12–15 participants. In Study 2, they will be delivered individually using virtual reality. The HIFT intervention protocol will be based on Cross-Training workouts, emphasizing high-intensity interval training combined with functional resistance movements. The QG intervention, a traditional Chinese practice, will include the Baduanjin sequence, one of the oldest and most beneficial practices for physical and mental health within the Qigong tradition. The MBSR intervention is entirely safe and includes

meditation, relaxation, and yoga techniques, following the official MBSR program developed by Kabat-Zinn. These interventions do not pose any health risks to participants. However, if a participant experiences any discomfort, pain, or issues during the intervention or study, they may speak with the principal investigator or medical researchers to rule out any risks to their continued participation.

Virtual Reality
Regarding the use of virtual reality devices, appropriate safety measures will be taken to prevent any risks. Participants will receive instructions on how to properly adjust the VR headset. They will also be informed to remove the headset for 30 seconds halfway through each session. Although no difficulties are expected with the device's controls or functionality due to the participants' age, the research team will remain vigilant to ensure smooth progress. Instructions and prompts will be carefully managed to avoid problems.

Detailed Information Provided to Participants
Participants will be clearly and thoroughly informed about the characteristics and objectives of the project and must complete and sign informed consent forms before continuing in the study.

Before each test, participants will receive detailed information about the objective and duration. After neuropsychological and MRI assessments, a written report will be provided to the participant.

Clinical, neuropsychological, and physical assessments will always be carried out by specialized staff at the Faculty of Psychology of the University of Barcelona. MRI scans will be performed at the Diagnostic Imaging Center of Hospital Clínic and Provincial de Barcelona.

Scheduling of various tests will be arranged by phone by a member of the research team, in order to coordinate convenient dates and times for participants.

The entity responsible for the processing of personal data is the General Secretariat of the University of Barcelona (CIF Q0818001J, Gran Via de les Corts Catalanes, 585 – 08007 Barcelona).

To ensure data confidentiality and security, the following measures will be taken:

- Data Storage: Secure platforms authorized by the University of Barcelona will be used, including OneDrive Cloud, Microsoft Forms, and Qualtrics.

- Protection of Participant Identity: Data will be pseudonymized. This means that although each participant's data will be linked to a unique code, personally identifiable information (such as names or emails) will be stored separately and accessed only by the principal investigator.

Research Team Commitments
The research team commits to the following for each participant:

- Maintain the confidentiality of the data collected, ensuring that after the first interview, participants are identified only by name and code for anonymization from the perspective of other researchers.
- Guarantee participants' right to withdraw from the study at any time without consequences.
- Clearly explain the results of the neuropsychological and MRI tests if relevant to the participant.
- Use the collected data exclusively for the purposes of this study. Neuropsychological, microbiota, and MRI data will not be shared or used for other projects without prior approval from the University of Barcelona's Bioethics Commission.
- Adhere to the Commission's communications on ethical concerns in scientific publishing and participant information sheets.
- Each participant will receive €30 as financial compensation.
- Ensure that no form of plagiarism is committed in the research content by any team member, in accordance with codes of good research practice.

Informed Consent from Study Participants

Each participant in Study 1 or 2 will sign the appropriate informed consent form attached to the documentation.

To this end, the principal investigator signs this document in Barcelona, on September 20, 2023.

Dr. Maria Mataró
Principal Investigator of the Project

UNIVERSITY OF BARCELONA

BIOETHICS COMMISSION

Participant Information Sheet

Active Youth, Healthy Mind: Strategies for Brain Health and Psychological Well-being in Young People (YoungFitT: Young Fitness Technology). Study 1.

1. INTRODUCTION AND PURPOSE OF THE STUDY

The main objective of this study is to investigate the effects of different mind-body modalities—specifically HIFT, Qigong, and MBSR—on brain health, cognition, and emotional well-being. The study also aims to explore the biological mechanisms underlying these effects and account for factors influencing individual variability in the brain's response. It is a collaborative project involving researchers from the University of Barcelona, Ramon Llull University, INEFC, and the Jordi Gol Primary Care Research Institute.

In the context of this research, we are asking for your collaboration in the study, as you meet the following inclusion criteria: being between 18 and 25 years old and currently enrolled in university studies. This collaboration involves first participating in an evaluation phase, followed by an intervention phase, and then a post-intervention evaluation phase, as explained below:

– Baseline evaluation of cognitive function, emotional well-being, physical condition, microbiota, and neuroimaging.

1. Collection of personal sociodemographic data (age, sex and gender, etc.) and health background.
2. Neuropsychological assessment. This is a brief test lasting approximately one and a half hours, during which various functions will be evaluated, such as memory, attention, language, executive functions, and information processing speed, in order to obtain a detailed profile of your cognitive status. Additionally, questionnaires will be administered to assess emotional state, stress, sleep, and quality of life, among other factors.
3. Assessment of physical condition and fitness through physical tests lasting about one hour.
4. Microbiota analysis. You will be asked to provide a small stool sample for microbiota analysis.
5. Structural and functional magnetic resonance imaging (MRI). MRI poses no health risk, as it is a completely safe technique. However, it may cause some discomfort due to noise or confinement, such as claustrophobia or anxiety during the scan. This technique is contraindicated for individuals with certain metal implants or devices, such as cardiac or ear prostheses or pacemakers. If any participant has such implants, the case will be evaluated individually. All MRIs will be reviewed by a neuroradiologist. In case of any clinically significant findings, the participant will be informed and will be able to collect the report and images at the center. The aim of this test is to examine the structure and function of different brain areas before and after the interventions, offering a highly detailed view of how the interventions affect the brain and how these effects relate to cognitive and emotional benefits.

Both MRI and microbiota analysis will be conducted randomly on a selected group of participants.

– Participation in the study on the effects of three interventions.

You will be randomly assigned to one of the following intervention groups: Mindfulness-Based Stress Reduction (MBSR), Qigong (QG), or High-Intensity Functional Training (HIFT). The interventions will last for 12 weeks and will be conducted online in groups of 12–15 participants. The HIFT and QG interventions will include three weekly sessions: two 60-minute group sessions conducted online by experts, and one individual self-guided session. The MBSR intervention will follow the official MBSR program designed by Kabat-Zinn, with some adaptations, and will consist of three sessions per week: one 120-minute live online session and two 20-minute sessions. At the beginning of the interventions, you will receive an introductory session and a written manual containing safety guidelines to prevent injuries or other possible adverse experiences.

– Post-intervention evaluation.

Cognitive, emotional well-being, and physical condition assessments will also be carried out after the interventions. For the relevant participants, microbiota analysis and magnetic resonance imaging (MRI) will be conducted. A follow-up will take place 12 weeks after the end of the study, during which participants will complete online questionnaires assessing their physical and psychological well-being. At the end of the program, a subset of participants will be invited to take part in a qualitative interview.

2. RISKS AND BENEFITS OF THE STUDY. Participation in this study poses no risk to your health, as the procedures to be performed are completely harmless.
On the contrary, it offers important benefits both for you and for society, as it aims to determine the effects of these interventions on brain health, as well as on your cognitive, emotional, and general health status, and to investigate the mechanisms and factors involved.

3. WITHDRAWAL FROM THE STUDY. Your participation is entirely voluntary, and you are completely free to decline participation or withdraw from the study at any time, without this decision resulting in any negative consequences.

4. CONFIDENTIALITY AND DATA PROTECTION. Only the principal investigator will have access to your identity. The General Secretariat of the University of Barcelona is, in accordance with personal data protection regulations, the legal entity responsible for processing your data within the framework of this project. This does not mean that the Secretariat will have access to your identity, the data generated, or the informed consent form—except in cases of legal

obligation (for example, if someone files a complaint with the data protection authority or before a judge or court). In accordance with the European Union's General Data Protection Regulation (GDPR), we inform you that the contact details of the General Secretariat are: Gran Via de les Corts Catalanes, 585, 08007 Barcelona**,** Email: . If at any point you wish to exercise the rights granted to you by data protection regulations (including the right to access your data, request its rectification, deletion, objection, portability, or limitation of processing), you must attach a copy of your ID card or another valid identification document. The personal data collected will be used solely for the purpose of managing and carrying out the YoungFitT research project, based on your consent, which you may revoke at any time without retroactive effect. The recipients of the personal data are the University itself, specifically the project's research team, and, where applicable, any entities in charge of data processing. No data will be shared with third parties unless required by law. These personal data will be retained until the project's objectives have been achieved and its results have been published (approximately up to 5 years after the end of the project). If you believe your rights have not been properly respected, you may contact the University of Barcelona's Data Protection Officer by postal mail (Gran Via de les Corts Catalanes, 585, 08007 Barcelona) or by email . You may also file a complaint with the Catalan Data Protection Authority: https://apdcat.gencat.cat/ca/inici**.** This study complies with Regulation (EU) 2016/679 of the European Parliament and of the Council of 27 April 2016 on the protection of natural persons regarding the processing of personal data and the free movement of such data (which repealed Directive 95/46/EC – General Data Protection Regulation), as well as Organic Law 3/2018 of 5 December, on the protection of personal data and the guarantee of digital rights.

5. COMPENSATION. Participation in the study includes a financial compensation of €30.

6. RIGHT TO ACCESS RESULTS. At the end of the study, if you wish, you will be able to access the results of your own participation and the overall study findings.

7. SIGNATURE IN DUPLICATE

The study participation document will be signed in two identical copies, and one printed copy will be given to the participant.

8. QUESTIONS AND INQUIRIES. Participants have the right to ask any questions they consider relevant regarding the characteristics of the study and their own participation in it. For any questions or clarifications related to the study, you may contact the research team. Below is the contact information: Principal Investigator of the Study: Dr. Maria Mataró**,** Professor of Psychobiology, University of Barcelona.

UNIVERSITY OF BARCELONA

BIOETHICS COMMISSION

Contact numbers: 638 273 131 (Project-specific phone). (Neuropsychology Laboratory, Faculty of Psychology, University of Barcelona).

a) In accordance with the aforementioned regulation, the University of Barcelona (with tax ID CIF Q0818001J and registered address at Gran Via de les Corts Catalanes, 585 – 08007 Barcelona), as the entity responsible for processing personal data, informs you that you may contact the Data Protection Officer either by postal mail (Travessera de les Corts, 131–159, Pavelló Rosa, 08028 – Barcelona) or by email

b) You have the right to access your data, request the rectification of inaccurate data, or, if applicable, request its deletion. You also have the right to restrict its processing, object to it, and withdraw your consent for specific purposes. These rights may be exercised by submitting a written request to the postal or email address provided in the previous paragraph. Additionally, you are informed of your right to file a complaint with the Catalan Data Protection Authority if you believe that any action by the University of Barcelona infringes upon your rights.

UNIVERSITY OF BARCELONA

BIOETHICS COMMISSION

INFORMED CONSENT OF THE PARTICIPANT

Title of the research project: Active Youth, Healthy Mind: Strategies for Brain Health and Psychological Well-Being in Young People (YoungFitT: Young Fitness Technology). Study 1.

The volunteer must read and answer the following questions carefully: (Please circle the answer you consider correct)

Have you read all the information provided to you about this project?	YES / NO
Have you had the opportunity to ask and discuss questions about the project?	YES / NO
Have you received sufficient information about this project?	YES / NO
Have you received satisfactory answers to all your questions?	YES / NO
Which researcher informed you about this project? (name and surname):	YES / NO
Do you understand that you are free to withdraw from this project without any consequences?	YES / NO
At any time:	YES / NO
Without giving any reason:	YES / NO
Do you understand the possible risks associated with your participation in this project?	YES / NO
Do you agree to participate?	YES / NO
Do you agree to participate in the MRI scan and microbiota analysis if selected?	YES / NO
I understand that I may be asked to participate in a qualitative interview.	YES / NO
Will you receive any compensation for participating?	YES / NO
Do you consent to your personal data being processed as described?	YES / NO

Signature: .... **Date:** ....

UNIVERSITY OF BARCELONA

BIOETHICS COMMISSION

Full name of the volunteer: ....

If at any time in the future you have questions or comments about this project, or if you wish to withdraw your participation, please contact: Dr. Maria Mataró. Professor of Psychobiology. Dept. of Clinical Psychology and Psychobiology. Faculty of Psychology. Pg. de la Vall d'Hebron 171. 08031 Barcelona. Contact email:
Phone numbers:

Place, date, and signature of the researcher: ....

Copy for the participant / Copy for the researcher

a) In accordance with the aforementioned regulation, the UNIVERSITY OF BARCELONA (with CIF Q0818001J and registered office at Gran Via de les Corts Catalanes, 585 - 08007 Barcelona), as the data controller, informs you that you may contact the Data Protection Officer by written request at the postal address (Travessera de les Corts, 131-159, Pavilion Rosa, 08028 - Barcelona), or by email

b) You have the right to access your data, request the rectification of inaccurate data or, if applicable, request its deletion, as well as to restrict its processing, object to it, and withdraw your consent for certain purposes. You may exercise these rights by writing to the postal address or by sending an email to the address mentioned above. Additionally, we inform you of your right to lodge a complaint with the Catalan Data Protection Authority if you consider that any action by the University of Barcelona infringes upon your rights.

INFORMATION SHEET FOR PARTICIPANTS

Active Youth, Healthy Mind: Strategies for Brain Health and Psychological Well-being in Young People with Virtual Reality (YoungFitT: Young Fitness Technology). Study 2.

UNIVERSITY OF BARCELONA

BIOETHICS COMMISSION

1. INTRODUCTION AND PURPOSE OF THE STUDY

This study aims to investigate the effects of different mind-body modalities performed in a virtual reality (VR) environment—specifically HIFT-VR (High-Intensity Functional Training), QG-VR (Qigong), and MBSR-VR (Mindfulness-Based Stress Reduction)—on brain health, cognition, and emotional well-being in university students. Additionally, it will explore underlying biological mechanisms and consider factors that may influence individual differences in young brains' responses to these exercise modalities.

This project involves coordinated participation of researchers from the Department of Clinical Psychology and Psychobiology of the Faculty of Psychology at the University of Barcelona, the Faculty of Psychology at Ramon Llull University – Blanquerna, the National Institute of Physical Education of Catalonia (INEFC), and the Jordi Gol Primary Care Research Institute.

You are invited to participate because you meet the inclusion criteria: aged between 18 and 25 and currently enrolled in university studies. Your participation involves three phases: a baseline assessment, an intervention phase, and a post-intervention assessment.

Baseline Assessment:

1. Collection of sociodemographic data (age, sex, gender, etc.) and health background.
2. Neuropsychological assessment: A brief session (~1.5 hours) evaluating memory, attention, language, executive functions, and information processing speed to determine your cognitive profile. Emotional status, stress, sleep, and quality of life questionnaires will also be administered.
3. Physical condition assessment: Physical fitness tests lasting about 1 hour.

Participation in the Intervention Phase:

You will be randomly assigned to one of the following VR-based intervention groups: MBSR-VR, QG-VR, or HIFT-VR. The interventions will last 12 weeks and be conducted autonomously using virtual reality. HIFT-VR and QG-VR include three one-hour weekly sessions. MBSR-VR follows the official Kabat-Zinn MBSR program, adapted to three days per week.

Participants will perform training while wearing virtual reality goggles. At the start of the program, you'll receive an information session and a safety manual to prevent injuries or other adverse experiences.

Post-Intervention Assessment: Cognitive, emotional well-being, and physical condition assessments will also be conducted after the interventions. A 12-week follow-up includes online questionnaires evaluating physical and psychological well-being. A subset of participants will be invited to take part in a qualitative interview.

2. RISKS AND BENEFITS. Participation involves no health risks, as all assessments are safe. The study provides benefits to you and society by identifying the effects on brain health, cognition, emotional well-being, and underlying factors.

3. WITHDRAWAL FROM THE STUDY. Your participation is voluntary. You are free to decline or withdraw at any time without consequence.

4. CONFIDENTIALITY AND DATA PROTECTION. Only the principal investigator will have access to your identity. The General Secretariat of the University of Barcelona is, with regard to compliance with personal data protection regulations, the legal entity responsible for processing this data within the framework of the project. This does not mean that the Secretariat has access to your identity, the data generated, or the informed consent form, except in cases of legal obligation (for example, if someone files a complaint with the data protection authority or before a judge or court). In accordance with the General Data Protection Regulation of the European Union, we inform you that the contact details of the General Secretariat are: Gran Via de les Corts Catalanes, 585, 08007 Barcelona; email: . You may contact them at any time to exercise your rights under data protection regulations (you may access your data and request its rectification, deletion, opposition, portability, or restriction). If you wish to exercise these rights, you must attach a copy of your ID or another valid document that verifies your identity. The personal data collected will only be used for the purpose of managing and executing the YoungFitT research project according to your consent, which you may revoke at any time without retroactive effect. The recipients of the personal data are the University itself—specifically, the project's research team and, if applicable, data processing service providers. No data will be disclosed to third parties unless required by law. This personal data will be retained until the project's objectives have been achieved and its results have been published (approximately up to 5 years from the end of the project). If you believe that your rights have not been properly addressed, you may contact the Data Protection Officer of the University of Barcelona by postal mail (Gran Via de les Corts Catalanes, 585, 08007 Barcelona) or by email . You may also file a complaint with the Catalan Data Protection Authority (https://apdcat.gencat.cat/ca/inici). This study complies with the "Regulation (EU) 2016/679 of the European Parliament and of the Council of 27 April 2016 on the protection of natural persons with regard to the processing of personal data

and on the free movement of such data, and repealing Directive 95/46/EC (General Data Protection Regulation)" as well as with "Organic Law 3/2018, of 5 December, on the Protection of Personal Data and the Guarantee of Digital Rights."

5. COMPENSATION. Participation will be compensated with 30€.

6. RIGHT TO KNOW THE RESULTS. You may request the results of your participation and the overall study.

7. SIGNATURE AND COPIES. This document will be signed in duplicate. One copy will be given to the participant.

8. QUESTIONS AND QUERIES. Participants may ask any questions regarding the study. For further information, contact: Dr. Maria Mataró, Professor of Psychobiology, University of Barcelona. Email: , Tel:

a) In accordance with the provisions of the aforementioned regulation, the University of Barcelona (with tax identification number CIF Q0818001J and registered office at Gran Via de les Corts Catalanes, 585 – 08007 Barcelona), as the entity responsible for processing personal data, informs you that you may contact the Data Protection Officer by sending a written request to the following postal address: Travessera de les Corts, 131–159, Pavelló Rosa, 08028 – Barcelona, or by email

b) You have the right to access your data, request the rectification of inaccurate data, request the deletion of your data where applicable, as well as to restrict its processing, object to it, and withdraw your consent for specific purposes. These rights can be exercised by writing to the postal or email address mentioned above.
Furthermore, you are informed of your right to file a complaint with the Catalan Data Protection Authority in case of any action by the University of Barcelona that you believe infringes your rights.

INFORMED CONSENT OF THE PARTICIPANT

Title of the research project: Active Youth, Healthy Mind: Strategies for Brain Health and Psychological Well-being in Young People with Virtual Reality (YoungFitT: Young Fitness Technology). Study 2.

The volunteer must read and answer the following questions carefully: (Please circle the answer you consider correct)

UNIVERSITY OF BARCELONA

BIOETHICS COMMISSION

Have you read all the information provided to you about this project?	YES/ NO
Have you had the opportunity to ask questions and discuss issues about the project?	YES/ NO
Have you received sufficient information about this project?	YES/ NO
Have you received satisfactory answers to all your questions?	YES/ NO
Which researcher explained this project to you? (Full name):	YES/ NO
Have you understood that you are free to withdraw from this project without any consequences?	YES/ NO
- At any time	YES/ NO
- Without giving any reason	YES/ NO
Have you understood the possible risks associated with your participation in this project?	YES/ NO
Do you agree to participate?	YES/ NO
I understand that I may be asked to participate in a qualitative interview	YES/ NO
Will you receive any compensation for participating?	YES/ NO
Do you consent to your personal data being processed as indicated?	YES/ NO

Signature: .... **Date:** ....

Full name of the volunteer: ....

If at any time in the future you wish to ask a question or make a comment about this project, or if you wish to withdraw your participation, please contact:
Dr. Maria Mataró. Professor of Psychobiology. Dept. of Clinical Psychology and Psychobiology. Faculty of Psychology. Pg. de la Vall d'Hebron 171. 08031 Barcelona.
Contact Email: . Contact phone numbers:

Place, date and signature of the researcher: ....

UNIVERSITY OF BARCELONA

BIOETHICS COMMISSION

Copy for the participant / Copy for the researcher

a) In accordance with the provisions of the aforementioned regulation, the UNIVERSITY OF BARCELONA (with CIF Q0818001J and registered office at Gran Via de les Corts Catalanes, 585 - 08007 Barcelona), as the data controller for personal data, informs you that you may contact the Data Protection Officer by sending a letter to the postal address (Travessera de les Corts, 131-159, Pavelló Rosa, 08028 - Barcelona), or via email

b) You have the right to access your data, request rectification of inaccurate data or, if applicable, request its deletion, as well as to limit its processing, object to it, and withdraw your consent for specific purposes. These rights may be exercised in writing to the postal address or via email mentioned in the previous paragraph. Furthermore, you are informed of your right to file a complaint with the Catalan Data Protection Authority if you believe that any action by the University of Barcelona has violated your rights.
