# Supplementary material for: The YoungFitT project: Study protocol for a randomized mixed-methods trial of physical exercise and mind-body interventions, with or without virtual reality, in university students
Source: PLoS One. 2025 Aug 1;20(8):e0328538. doi: 10.1371/journal.pone.0328538 (PMC12316210; doi:10.1371/journal.pone.0328538)
Supplement: S4 File — This focus group guide is used to explore user experience, technical considerations, motivation, and feedback mechanisms related to integrating HIFT, QG, and mindfulness into VR. The guide includes thematic sections and open-ended questions to support in-depth discussion and user-driven design. (PDF) [file pone.0328538.s004.pdf]

1. Introduction

"Welcome, and thank you for participating in this focus group session. Our goal is to explore how well-being practices such as High-Intensity Functional Training (HIFT), Mindfulness-Based Stress Reduction (MBSR), and Qigong can be effectively integrated into a VR environment. We are interested in your insights on engagement, usability, technical considerations, and overall experience. With your consent, this session will be recorded and transcribed for analysis. Your responses will remain anonymous, and all data will be used solely for research purposes. You are encouraged to share your honest opinions and experiences."

2. Focus Group Discussion Questions

2.1. General Experience and Adaptation of Well-being Practices to VR

- How do you feel about adapting well-being practices such as HIFT, mindfulness, and Qigong to VR?
- What challenges do you foresee in integrating these practices into a VR environment?
- What aspects of these activities work well in their original format that should be retained in VR?

2.2. VR Experience & Interaction

- What prior experiences do you have with VR?
- What technical features are critical to creating an immersive and user-friendly VR experience?
- How important is social interaction in VR well-being practices (individual vs. group engagement)?

- What aspects of VR (e.g., visuals, sounds, feedback) would help make participants feel engaged and comfortable?

2.3. Motivation and Adherence to VR Well-being Practices

- What factors would motivate users to continue practicing well-being activities in VR?
- How can we ensure engagement over a long-term intervention period?
- What role could gamification play in maintaining motivation, and what incentives should be included?

2.4. Customization and Sensory Experience in VR

- How important is it for users to be able to customize their VR experience (e.g., selecting environments, adjusting sound settings)?
- What elements (e.g., visuals, audio, haptic feedback) would enhance relaxation and immersion in VR-based mindfulness and Qigong sessions?
- How should sensory feedback (e.g., lighting adjustments, soundscapes, movement guidance) be integrated?

2.5. Technical Challenges and Accessibility

- What technical barriers might prevent users from engaging fully in VR well-being interventions?
- What accessibility concerns (e.g., motion sickness, hardware limitations, cost) should be considered?
- How can we ensure the intervention remains inclusive and accessible to a wide range of users?

2.6. Integration of Learning and Feedback Mechanisms

- How should theoretical content (e.g., mindfulness principles, exercise guidance) be integrated into the VR experience?
- Would real-time corrective feedback be beneficial in VR well-being activities? If so, how should it be implemented?
- What type of feedback (visual, auditory, haptic) is necessary to guide users in performing exercises correctly?

2.7. Evaluating VR Well-being Practices and Defining Success

- How will we determine whether the VR intervention is effective and engaging?
- What usability or experience assessments should be included to validate the system?
- How will we know when we have reached an optimal VR design (e.g., thematic saturation, participant consensus, usability testing results)?

3. Closing and Final Reflections

"Thank you for your valuable insights and participation. Before we conclude, do you have any final thoughts or recommendations on how to improve the VR well-being experience? Your feedback will directly inform the development of this intervention, ensuring it meets the needs of future users. If you have any additional suggestions after this session, feel free to reach out. We truly appreciate your time and contribution."
