# Supplementary material for: The YoungFitT project: Study protocol for a randomized mixed-methods trial of physical exercise and mind-body interventions, with or without virtual reality, in university students
Source: PLoS One. 2025 Aug 1;20(8):e0328538. doi: 10.1371/journal.pone.0328538 (PMC12316210; doi:10.1371/journal.pone.0328538)
Supplement: S5 File — This interview guide is designed to explore participants’ experiences, perceptions, and reflections related to the intervention. It includes open-ended questions covering engagement, motivation, perceived effects, barriers, and long-term impact. (PDF) [file pone.0328538.s005.pdf]

Introduction

"Before we begin, I want to remind you that this conversation is strictly confidential, and all data will be handled anonymously. The purpose of this interview is to better understand your experiences and perceptions of the intervention. To ensure accuracy and facilitate analysis, I'd like to ask for your permission to record this interview. Is that okay with you?"

(If the participant agrees, proceed).

1. General Experience with the Intervention

- Can you describe your overall experience with the program?
- What were the most positive aspects of your participation?
- Were there any challenges or difficulties you encountered?

2. Expectations and Motivation

- What were your initial expectations when joining this study?
- Did your expectations change over time? If so, how?
- What motivated you to participate, and did that motivation evolve throughout the intervention?

3. Engagement and Adherence

- How consistently were you able to participate in the sessions?
- What factors influenced your ability to stay engaged?
- Were there any barriers that made participation difficult? How did you manage them?

4. Perceived Effects of the Intervention

- What changes—physical, mental, or emotional—have you noticed in yourself since starting the program?
- Did the intervention impact any specific areas of your life, such as stress management, focus, or energy levels?
- Would you attribute these changes specifically to the intervention, or do you think other external factors played a role?

5. Social and Environmental Influences

- Did the group setting affect your experience? If so, in what way?
- Have friends, family, or colleagues noticed any changes in you since participating?
- Did you feel supported by the people around you in maintaining your participation?

6. Barriers and Opportunities for Improvement

- Were there any aspects of the intervention that you feel could be improved?
- What would have made it easier for you to participate more consistently?
- Would you have preferred a different format (e.g., in-person vs. virtual)? Why or why not?

7. Long-Term Impact and Sustainability

- Do you see yourself continuing any of the practices you learned in this intervention? Why or why not?
- What challenges do you anticipate in maintaining these practices independently?
- What type of support or resources would help you sustain these changes in the long term?

8. Broader Reflections and Future Research

- Based on your experience, what aspects of similar interventions do you think should be studied further?

- Do you have any recommendations for improving future programs like this?
- Is there anything else about your experience that we haven’t covered but that you feel is important to share?

Closing Statement

"Thank you for taking the time to share your experiences. Your insights are incredibly valuable for understanding the effects of this intervention. If you have any questions or concerns in the future, feel free to reach out. We appreciate your participation."
